# Latitude affects Morningness-Eveningness: evidence for the environment hypothesis based on a systematic review

**DOI:** 10.1038/srep39976

**Published:** 2017-01-03

**Authors:** Christoph Randler, Arash Rahafar

**Affiliations:** 1 University of Tubingen, Department of Biology, Auf der Morgenstelle 24, D-70726 Tubingen, Germany

## Abstract

Morningness-eveningness (M/E) is an individual trait related to a person’s sleep-wake cycle and preference for morning or evening hours. The “environment hypothesis” suggests that M/E is dependent on environmental factors, such as latitude, mean average temperature and photoperiod. We here analyzed a large number of datasets to assess this effect based on a systematic review. Data were from a total of 87 datasets and 35,589 individuals based on 28 countries. Partial correlations correcting for age revealed significant relationships between M/E and latitude, mean yearly temperature, photoperiod and sunset. Evening orientation was related to higher latitude, longer days and later sunset. Morning orientation was related to higher average temperatures. Percentage of females and sunrise time had no significant influence. These variables (sunset, temperature, photoperiod) were then input in a general linear model. The full model showed an influence of age and of sunset on CSM scores, but not of photoperiod and average temperature. Sunset, therefore, seems to be the most important statistical predictor for the observed latitudinal gradient.

Morningness-eveningness (M/E) preference is an individual trait related to a person’s sleep-wake cycle and preference for morning or evening hours^1^. Some people go to bed early and get up early while others prefer later bed and rise times. In addition, the feeling after awakening (drowsiness versus freshness) is another aspect, as are the preferences for specific hours for cognitive and physical peak performance. Basically, there are age-related changes in M/E, especially during puberty^2^, but also during adulthood, when people become more morning oriented with an increasing age^3^. Women are on average more morning oriented than men^4^,^5^.

The light-dark cycle (as manifested in sunrise, sunset and photoperiod) as well as other environmental cues (e.g., temperature) have been proposed to influence M/E^1^ and this was explicated as the “environment hypothesis”^6^. Latitude, as a surrogate was therefore used as an influential factor in some studies, because latitude is grossly correlated with both, average temperature and photoperiod. However, only a few studies have addressed this question in large scale studies or by country comparisons, and most country comparisons are based on few – but often contrasting – countries to show this effect. Randler^7^ assessed 17 countries but the sample was based on school pupils. There was an interesting effect with adolescents in the tropics being the earliest, and adolescents in the subtropics being the latest in M/E. Within the central European Time Zone, there was a gradient from Southwest (SW) to Northeast (NE) with a higher morning orientation towards the East and North. As this study was based on adolescents, there were interaction effects between age and morningness-eveningness because of the dramatic changes in M/E during adolescence^2^. Therefore, the environment hypothesis should be challenged in adults. A general latitudinal trend was proposed by some authors, e.g., within a country (Russia; Borisenkov *et al*.^8^; Brazil: Miguel *et al*.^9^; USA: White & Terman^10^) and between countries (Randler^7^; Sani *et al*.^11^). A higher evening orientation was found with an increasing distance from the equator^7^,^8^,^9^,^11^. Contradictory results have been found across the USA, where a higher morningness was related to higher latitudes^10^. However, a detailed analysis across a large number of countries has never been carried out.

The light regime should also have an influence on M/E. Sunrise, for example, seems to have an influence on M/E with people in the eastern parts of a time zone being more morning oriented. This was found in Germany^12^, Russia^8^ and in Turkey^13^. Also, when comparing Italy and Spain, Spaniards showed a higher eveningness^14^. However, such an effect was not found when comparing Germans and Poles^15^. Borisenkov *et al*.^8^ reported a correlation between sunrise and M/E with earlier sunrise related to higher morningness (see also Walch *et al*.^5^) A similar longitudinal effect was found in another animal species, in dogs (*Canis familiaris*^16^). Accordingly, sunrise and sunset are related to both latitude and longitude, and should be a better measure for the environmental influence of the sunlight.

Some authors further suggested that the differences between countries in M/E may be also related to the climate^7^,^17^,^18^, and people living in warmer climates should be more morning oriented. This is backed up by a study on birds (*Parus major*) where the chronotype was slightly earlier under higher temperature^19^.

Although social demands cannot be ruled out^5^,^20^, this present study sought evidence for the environment hypothesis in the “hard wired” factors of environmental variables. Here, we assess the influence of latitude on M/E across a wide sample of studies, and further test competing hypotheses, namely the influence of light versus temperature. To specify the environment hypothesis, some hypotheses were explicated:

1. Latitude should be correlated with M/E across a large number of studies, the farther away from the equator, the more evening orientation^7^,^9^.
2. Temperature was proposed as the key factor by Smith *et al*.^17^ and Tonetti *et al*.^18^, and these authors suggested that people living in the warmer climates may be more shifted towards morningness.
3. Photoperiod and seasonal variability of the photoperiod differ, with longer photoperiods in the North, leading to more evening orientation.
4. Sunrise and sunset times are related to M/E, with usually earlier sunrise times and later sunset times in the North during the summer. Earlier sunrise should be linked to morning preference^21^,^22^.
5. Given the human origin out-of-Africa, humans originated from a relatively stable light-dark cycle near the equator and settled towards northern latitudes. One could hypothesize that the variability of the sleep-wake cycle might be more stable near the equator. Then variance as measured by SD should increase with the distance to the equator.

## Results

Descriptive statistics are depicted in Tables 1 and 2. Data were from a total of 87 datasets and 35,589 individuals based on 28 countries. Bivariate partial correlations correcting for age revealed significant relationships between CSM scores and latitude, mean yearly temperature, photoperiod and sunset (Table 3). Evening orientation was related to higher latitude, longer photoperiod and later sunset. Morning orientation was related to higher yearly average temperatures. Percentage of females and sunrise time had no significant influence and were dropped from subsequent analyses. The SD of the CSM scores was unrelated with all variables (Table 3). After having shown the significant relationship between latitude and M/E, more detailed analyses were used to assess which of the independent variables is the most important predictor of CSM scores. The significant independent variables (sunset, temperature, photoperiod) from the bivariate correlations were input in the general linear model (Table 4). The full model showed an influence of age and of sunset on CSM scores, but not of photoperiod and average temperature (Table 4). Sunset, therefore, seems to be the most important statistical predictor for the observed latitudinal gradient.

## Discussion

This is the first large scale study that shows a latitudinal effect of M/E. Other studies were based on much smaller samples and countries. For example, Sani *et al*.^11^ was based on 5 countries and Smith *et al*.^17^ on 6 countries. Thus, the study confirms the environment hypothesis^6^ around the globe. Higher latitudes are associated with higher eveningness. However, within the USA, there was contradictory effect of people with dark irides having a higher morningness in the higher latitudes, but the sample was smaller (N = 191^10^), and we did not use iris colour in our analysis.

Temperature was also related to CSM scores, partially confirming the temperature hypothesis^18^. Similarly, photoperiod and sunset showed an association in the bivariate correlations. Sunrise was unrelated to M/E. Further detailed analyses, however, showed that it is the sunset time on the longest day of the year that showed the highest statistical influence. This is interesting because it shows that it is not sunrise as found in the study of Borisenkov^21^ but sunset. Time of sunrise was a stronger predictor of chronotype in Russia than the time of sunset and day length^21^. However, this could be owed to the different populations because Borisenkov’s^21^ study was a within country comparison, while this study is a between country comparison. Future studies might focus on assessing the variance in sleep-wake behaviour within and between countries simultaneously.

The variance in sleep-wake behaviour as measured by SD was uncorrelated to any of the independent variables. Although intuitively correct, the time since the human origin out of Africa may be long enough to have the human population adapted to the local and more oscillation light and temperature environments of the higher latitudes.

This study is based on an approach of using published data of CSM values and links those with the latitude of the respective studies. Another approach would be assessing effect sizes of the few published studies on latitude but these few studies (all cited in the Introduction) use different approaches (e.g., questionnaires, behavioural measures) as well as different latitudinal gradients, and are based on within and among country comparisons. However, when there are more studies available, a meta-analysis seems worthwhile.

Although social factors also have an influence on sleep-wake behaviour^5^ and they could not be ruled out in the current study, the correlation coefficients of 0.36 to 0.50 are quite strong for questionnaire data, rendering the environment hypothesis very likely. Previous studies commented on social factors, but these studies were mostly based on a very small number of countries.

As a conclusion, the environment hypothesis can be confirmed and even more specified with sunset being the most important predictor of M/E.

## Methods

This study is based on aggregated, published data and the study followed the protocol in Randler^3^ to locate all relevant studies that were published in the domain of M/E. The search was based on a systematic review following the PRISMA guidelines^23^.

**Figure 1:**
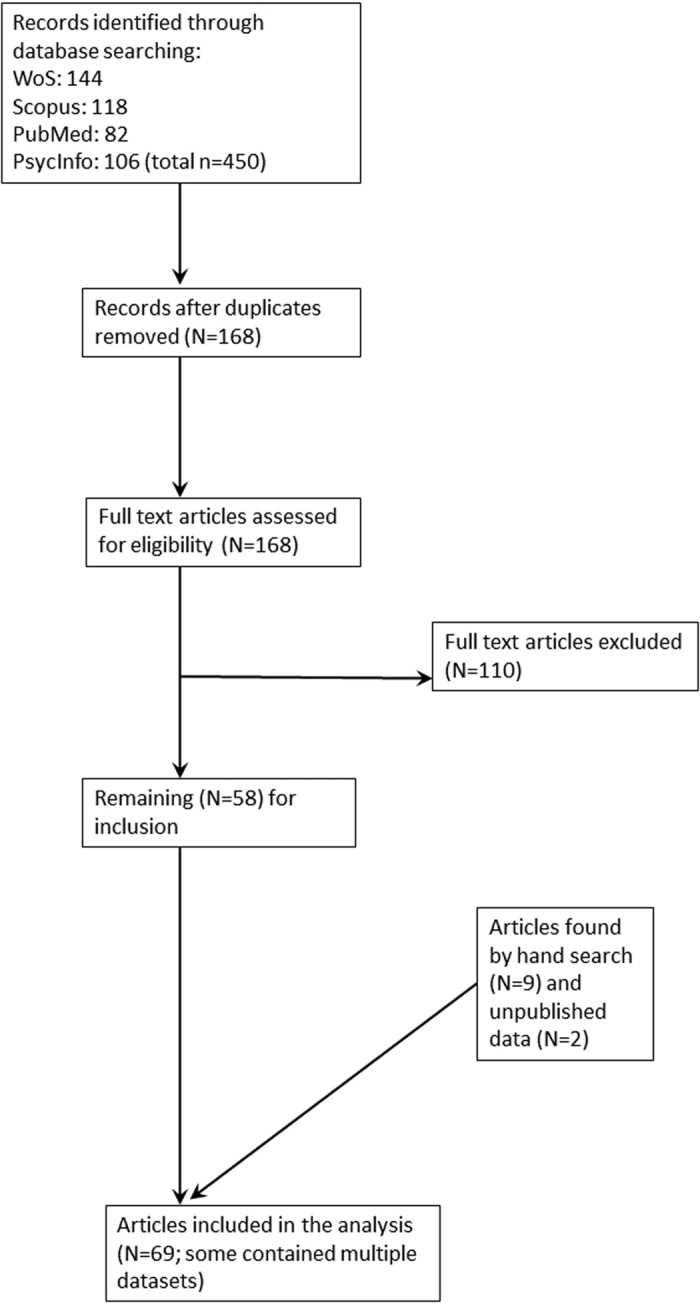
Flowchart for the included studies.

Initially, the terms “Composite” and “Scale” and “Morningness” in Scopus, Web of Science, PubMed, and PsychInfo were searched on 18.7.2015 (title, abstract, and keywords). A total of 450 records have been retrieved. After checking for doublets, 168 records remained (see Fig. 1). Additionally, papers were raised by hand search and by snowball system. Exclusion criteria were i) other questionnaires used than the CSM, ii) clinical samples, iii) shift workers, iv) school pupil samples were excluded because of the dramatic changes in sleep-wake cycle from early childhood to adulthood^2^. This was to avoid masking effects of the dramatic changes during adolescence; v) duplicate samples (that do not inevitably represent duplicate publications, when two different questions were addressed with the same sample), vi) samples smaller than N = 50. Studies from the Randler group were excluded but the database available was used and partitioned into four different age groups (18–20 years, 21–30 years, 31–50 years, and 51–79 years^3^). Concerning missing data, authors were contacted by e-mail based on the mail address given in the paper. Some authors provided additional unpublished data or data published in journals that were not detected by the search routine. After screening, 87 datasets remained in the study with a sufficient amount of information (Supplementary Tables 1 and 2). The basic requirements for this study was that the mean age was reported, as well as the mean CSM scores. In addition, the number of males and females were taken and the percentage of females was calculated or directly extracted from the articles. Differences to the search routine of ref. 3 and exclusion criteria are reported in Supplementary Material S3. Mainly, this difference in studies was because small sample studies have been excluded. Second, additional “hidden” studies, that were not detected by the search engines (see above) but are published, have been detected by hand search, and third, some author sent data upon request. Also, some samples have been recalculated (see information in S1). This has been done when samples included school pupils and the raw data where available. Then, based on our exclusion criterion iv) (see above), these cases have been removed. In addition, we matched some studies/samples that apparently are based on the same dataset and preferred samples with a clear location over samples where two different locations have been merged. Containing clinical samples, some studies presented data on the healthy control group. These studies have been used and in the comments column, we have indicated this (S1).

All studies were based on the Composite Scale of Morningness (CSM, Smith *et al*.^24^) for several reasons: (i) the CSM is the only questionnaire where cross-cultural comparisons for the factor structure have been made^25^, thus the CSM is the only questionnaire that has yet been tested psychometrically for cross-cultural comparisons; (ii) the CSM is mostly used in cross-country comparisons, and (iii) the CSM is a metric scale and the CSM score is used for comparisons, while other questionnaires are mainly based on categorizations. If different populations were reported in a study, all were treated separately.

Geographical data were extracted from Wikipedia. Latitude was coded into north of the equator (+) and south of the equator (−). Latitude was extracted when the original location was given (e.g. a city or its surroundings). If only a specific region of sampling was given, for example when the sampling was made online in a specific, geographically restricted area, we established the mean value of latitude, e.g., when studies were made at three locations that were nearby. Then, the mean between north and south of this geographic region was calculated. However, we excluded studies that were, e.g., online samples throughout the whole USA because this country is far too big to calculate a mean latitude value. When no detailed city was given but information lead to the conclusion that it was the University city where the authors worked, then these data were used. Afterwards, latitude was mirrored at the equator, so that both 20°N and 20°S received the value 20.

Mean yearly average temperature was extracted from http://www.climate-charts.com, www.weatherbase.com, and www.klimadiagramme.de (accessed May 2016). Sunrise, sunset and photoperiod data were extracted from: http://www.timeanddate.com for the 21^st^ of June for the Northern, and 21^st^ of December on Southern hemisphere to reflect sunlight during the longest day of the year (accessed June 2016).

### Statistical analysis

Only studies where the mean age and the mean CSM score could be obtained were used. Age has a significant influence on morningness-eveningness scores and, therefore, the mean age as reported in the studies was used as covariate^3^. As women and men differ in M/E, the percentage of females was used as a control variable, assuming that a higher percentage of women may shift the mean value to a higher value. First, bivariate correlations were used to establish the relationships. Then, different regression models were used to assess if the relationship between latitude and CSM scores is linear. Afterwards, general linear mixed models were used to assess the relationship of all independent variables (that have been found to be significant in the correlations) simultaneously. SPSS 23.0 was used for analysis.

## Additional Information

**How to cite this article**: Randler, C. and Rahafar, A. Latitude affects Morningness-Eveningness: evidence for the environment hypothesis based on a systematic review. *Sci. Rep.* **7**, 39976; doi: 10.1038/srep39976 (2017).

**Publisher's note:** Springer Nature remains neutral with regard to jurisdictional claims in published maps and institutional affiliations.

## Supplementary Material

Supplementary Information

## Figures and Tables

**Table 1:** Overview over studies and countries based on the Composite scale of Morningness (CSM) to assess chronotype/morningness.

Country	No. of studies
Argentina	2
Australia	5
Brazil	1
Canada	4
China/Hong Kong	1
Colombia	2
France	6
Germany	5
Hungary	1
India	5
Iran	1
Italy	3
Netherlands	1
Norway	2
Peru	1
Poland	2
Portugal	1
Romania	3
Russia	1
Singapore	1
Slovakia	1
South Korea	5
Spain	10
Tenerife	1
Thai	2
Turkey	3
UK	2
USA	15

**Table 2:** Descriptive statistics of the sample.

	N	Minimum	Maximum	Mean	SD
Latitude (mirrored)	87	1.00	64.00	38.71	11.79
Latitude	87	−38.00	64.00	32.83	23.77
yearly average temperature	87	2.40	28.40	13.87	5.78
sunrise	87	3:35	7:08	5:35	0:38
photoperiod	87	12:11	19:01	15:07	1:16
sunset	87	18:10	23:11	20:42	1:06
mean age	87	17.64	78.90	28.66	11.65
sample size	87	54	3340	421.78	460.37
Mean CSM scores	87	28.90	44.90	35.49	3.71
SD (CSM)	83	4.13	9.07	6.55	0.812
Percentage female	86	5.00	100.00	63.48	17.50

**Table 3:** Bivariate partial correlations (correcting for age) between CSM scores and different geographical variables (Latitude, average temperature, sunrise, sunset, and photoperiod).

		CSM score	SD CSM
Latitude_mirror	Pearson’s *r*	−0.418	0.162
	*p*	<0.001	0.148
Mean yearly average temp	Pearson’s *r*	0.360	−0.166
	*p*	0.001	0.139
sunrise	Pearson’s *r*	−0.117	0.048
	*p*	0.299	0.671
photoperiod	Pearson’s *r*	−0.361	0.087
	*p*	0.001	0.441
sunset	Pearson’s *r*	−0.490	0.129
	*p*	<0.001	0.249
Percentage female	Pearson’s *r*	−0.040	−0.034
	*p*	0.726	0.763

SD = standard deviation of the CSM score. Based on N = 81 studies for which all information was available.

**Table 4:** General linear mixed model with CSM score as dependent variable, mean age, mean yearly average temperature, photoperiod and sunset as independent variables.

Source	F-value	Significance
Constant	49.372	<0.001
Mean age	87.136	<0.001
Mean temperature	0.793	0.376
Photoperiod	2.422	0.124
Sunset	12.736	0.001

N = 87 studies.

## References

[b1] Adan A. . Circadian typology: A comprehensive review. Chronobiol. Int. 29, 1153–1175 (2002). 10.3109/07420528.2012.719971 23004349

[b2] Carskadon M. A., Vieira C. & Acebo C. Associations between puberty and delayed phase preference. Sleep 16, 258–262 (1993). 8506460 10.1093/sleep/16.3.258

[b3] Randler C. Ontogeny of morningness-eveningness across the adult human lifespan. Sci. Nat. 103(3), 1–4 (2016). 10.1007/s00114-015-1326-z 26715354

[b4] Randler C. Gender differences in morningness-eveningness assessed by self-report questionnaires: a meta-analysis. Pers. Individ. Dif. 43, 1667–1675 (2007).

[b5] Walch O. J., Cochran A. & Forger D. B. A global quantification of “normal” sleep schedules using smartphone data. Sci. Adv. 2(5), e1501705 (2016). 27386531 10.1126/sciadv.1501705 PMC4928979

[b6] Horzum M. B. . Morningness–eveningness and the environment hypothesis–A cross-cultural comparison of Turkish and German adolescents. Chronobiol. Int. 32(6), 814–821 (2015). 26061589 10.3109/07420528.2015.1041598

[b7] Randler C. Morningness-eveningness comparison in adolescents from different countries around the world. Chronobiol. Int. 25, 1017–1028 (2008). 19005902 10.1080/07420520802551519

[b8] Borisenkov M. F. Chronotype, sleep length, and school achievement of 11- to 23-year-old students in northern European Russia. Chronobiol. Int. 27, 1259–1270 (2010). 20653453 10.3109/07420528.2010.487624

[b9] Miguel M., de Oliveira V. C., Pereira D. & Pedrazzoli M. Detecting chronotype differences associated to latitude: a comparison between Horne–Ostberg and Munich Chronotype questionnaires. Ann. Human Biol. 41, 107–110 (2014). 10.3109/03014460.2013.832795 24059265

[b10] White T. M. & Terman M. Effect of iris pigmentation and latitude on chronotype and sleep timing. Chronbiol. Int. 20, 1193–1195 (2003).

[b11] Sani M. . Daily activity patterns of 2316 men and women from five countries differing in socioeconomic development. Chronobiol. Int. 32, 650–656 (2015). 26035482 10.3109/07420528.2015.1038559 PMC4769639

[b12] Roenneberg T., Kumar C. J. & Merrow M. The human circadian clock entrains to sun time. Curr. Biol. 17, 44–45 (2007). 10.1016/j.cub.2006.12.011 17240323

[b13] Masal E. . Effects of longitude, latitude and social factors on chronotype in Turkish students. Pers. Individ. Dif. 86, 73–81 (2015).

[b14] Adan A. & Natale V. Gender differences in morningness/eveningness preference. Chronobiol. Int. 19, 709–720 (2002). 12182498 10.1081/cbi-120005390

[b15] Jankowski K. S., Vollmer C., Linke M. & Randler C. Differences in sun time within the same time zone affect sleep-wake and social rhythms, but not morningness preference: Findings from a Polish-German comparison study. Time Soc. 23, 258–276 (2014).

[b16] Randler C., Díaz-Morales J. F. & Jankowski K. S. Synchrony in chronotype and social jetlag between dogs and humans across Europe. Time Soc. In press, doi: 10.1177/0961463X15596705.

[b17] Smith C. S. . Investigation of morning-evening orientation in six countries using the preferences scale. Pers. Individ. Dif. 32, 949–968 (2002).

[b18] Tonetti L., Sahu S. & Natale V. Circadian preference in Italy and India: A comparative study in young adults. Pers. Individ. Dif. 53, 355–358 (2012).

[b19] Lehmann M., Spoelstra K., Visser M. E. & Helm B. Effects of Temperature on Circadian Clock and Chronotype: An Experimental Study on a Passerine Bird. Chronobiol. Int. 29 **(8)**, 1062–1071 (2012). 22881370 10.3109/07420528.2012.707159

[b20] Lo J. C., Leong R. L. F., Loh K.-K., Dijk D.-J. & Chee M. W. L. Young adults’ sleep duration on work days: differences between East and West. Front. Neurol. 5(**81**), 1–12 (2015). 10.3389/fneur.2014.00081 PMC4036075 24904524

[b21] Borisenkov M. F. The pattern of entrainment of the human sleep-wake cycle by the natural photoperiod in the north. Chronobiol. Int. 28, 921–929 (2011). 22080737 10.3109/07420528.2011.623978

[b22] Shawa N. & Roden L. C. Chronotype of South African adults is affected by solar entrainment. Chronobiol. Int. 33(3), 315–323 (2016). 26953619 10.3109/07420528.2016.1144608

[b23] Liberati A. . The PRISMA statement for reporting systematic reviews and meta-analyses of studies that evaluate health care interventions: explanation and elaboration. Ann. Internal Med. 151(4), 65–94 (2009). 10.7326/0003-4819-151-4-200908180-00136 19622512

[b24] Smith C. S., Reilly T. C. & Midkiff K. Evaluation of three circadian rhythm questionnaires with suggestions for an improved measure of morningness. J. Appl. Psychol. 74, 728–738 (1989). 2793773 10.1037/0021-9010.74.5.728

[b25] Di Milia L., Adan A., Natale V. & Randler C. Reviewing the psychometric properties of contemporary circadian typology measures. Chronobiol. Int. 30, 1261–1271 (2013). 24001393 10.3109/07420528.2013.817415

