# Supplementary material for: Latitude affects Morningness-Eveningness: evidence for the environment hypothesis based on a systematic review
Source: Sci Rep. 2017 Jan 3;7:39976. doi: 10.1038/srep39976 (PMC5206670; doi:10.1038/srep39976)
Supplement: Supplementary Information [file srep39976-s1.pdf]

SUPPLEMENTARY INFORMATION

Latitude affects Morningness-Eveningness: evidence for the environment hypothesis based on a systematic review

Christoph Randler & Arash Rahafar

Supplement 1: Studies used for the analysis in alphabetical order and comments. The numbering of the studies follows the numbering in the data sheet (S3).

1. Adan, A., Natale, V., Caci, H., & Prat, G. (2010). Relationship between circadian typology and functional and dysfunctional impulsivity. *Chronobiology International*, 27, 606-619. --- Data retrieved from the authors.
2. Ahn, Y. M., Chang, J., Joo, Y. H., Kim, S. C., Lee, K. Y., & Kim, Y. S. (2008). Chronotype distribution in bipolar I disorder and schizophrenia in a Korean sample. *Bipolar Disorders*, 10(2), 271-275.
3. Arbabi, Tayebeh (2009). Personal data collection.
4. Bae, M., Lee, K., Baek, J. H., Kim, J. S., Cho, Y., Ryu, S., ... & Hong, K. S. (2014). Lifetime experiences of hypomanic symptoms are associated with delayed and irregular sleep–wake cycle and seasonality in non-clinical adult samples. *Comprehensive psychiatry*, *55*(5), 1111-1115.
5. Bhatia, T., Agrawal, A., Beniwal, R. P., Thomas, P., Monk, T. H., Nimgaonkar, V. L., & Deshpande, S. N. (2013). A Hindi version of the Composite Scale of Morningness. *Asian Journal of Psychiatry*, 6(6), 581-584. --- used as three different studies/datasets because of three different samples given in the paper.
6. Blagrove, M., & Akehurst, L. (2000). Personality and dream recall frequency: Further negative findings. *Dreaming*, 10(3), 139.
7. Bohle, P., Tilley, A. J., & Brown, S. (2001). Psychometric evaluation of the Early/Late Preferences Scale. *Ergonomics*, 44, 887-900.
8. Boudebesse, C., Lajnef, M., Geoffroy, P. A., Bellivier, F., Nieto, I., Gard, S., ... & Etain, B. (2013). Chronotypes of bipolar patients in remission: Validation of the French version of the circadian type inventory in the FACE-BD sample. *Chronobiology International*, *30*(8), 1042-1049.
9. Brown, F. M. (1993). Psychometric equivalence of an improved Basic Language Morningness (BALM) scale using industrial population within comparisons. *Ergonomics*, 36, 191-197.
10. Caci, H., Nadalet, L., Staccini, P., Myquel, M. & Boyer, P. (1999). Psychometric properties of the French version of the Composite Scale of Morningness. *European Psychiatry*, 14, 284-290.
11. Caci, H., Nadalet, L., Staccini, P., Myquel, M. & Boyer, P. (2000). The Composite Scale of Morningness: further psychometric properties and temporal stability. *European Psychiatry*, 15, 278-281.
12. Caci, H., Adan, A., Bohle, P., Natale, V., Pornpitakpan, C., & Tilley, A. (2005). Transcultural properties of the composite scale of morningness: the relevance of the “morning affect” factor. *Chronobiology International*, 22, 523-540.
13. Caci, H., Deschaux, O., Adan, A., & Natale, V. (2009). Comparing three morningness scales: Age and gender effects, structure and cut-off criteria. *Sleep Medicine, 10*, 240-245.
14. Di Milia L, Mueller H. (2012). Does impression management impact the relationship between morningness–eveningness and self-rated sleepiness? *Personality and Individual Differences, 52,* 702–706.

15. Díaz-Morales, J. F., & Sánchez-López, M. (2005). Composite scales of morningness and preferences: preliminary validity data in Peruvian undergraduates. *Ergonomics, 48,* 354-363.

16. Díaz-Morales, J.F., & Sánchez-López, M.P. (2004). Composite and preferences scales of morningness: reliability and factor invariance in adult and university samples. *The Spanish Journal of Psychology, 7(2*), 93-100.

17. Díaz-Morales, J.F. (2007). Morning and evening-types: exploring their personality styles. *Personality and Individual Differences,* 43, 769-778.

18. Díaz-Morales, J. F., & Sánchez-López, P. Mª. (2008). Morningness-Eveningness and Anxiety among Adults: A Matter of Sex/Gender? *Personality and Individual Differences, 44*, 1391-1401.

19. Díaz-Morales, J. F., & Aparicio, M. (2003). Relaciones entre estilos de personalidad y tipología circadiana. *Anales de Psicología, 19(2)*, 247-256.

20. Diaz-Ramirez (2000) unpublished PhD thesis, cited in Díaz Morales, J. F. & Sánchez-López M. P.(2005) Propiedades psicométricas de la Escala de Preferencias y la Escala Compuesta de Matutinidad. Psicothema 17, 471-477.

21. Digdon, N. L. (2010) Circadian preference and college students' beliefs about sleep education. *Chronobiology International*, 27, 297-317.

22. Digdon, N., & Landry, K. (2013). University students' motives for drinking alcohol are related to evening preference, poor sleep, and ways of coping with stress. *Biological Rhythm Research, 44*(1), 1-11.

23. Dresch, V., Sánchez López, M. D. P., & Aparicio García, M. E. (2005). Diferencias de personalidad entre matutinos y vespertinos. *Revista Latinoamericana de Psicología*, 37(3), 509-522.

24. Driscoll, H. C., Serody, L., Patrick, S., Maurer, J., Bensasi, S., Houck, P. R., ... & Reynolds, C. F. (2008). Sleeping well, aging well: a descriptive and cross-sectional study of sleep in "successful agers" 75 and older. *The American Journal of Geriatric Psychiatry,* 16(1), 74-82.

25. Gil, E., Abdo, P. L., Rodriguez, M., Zanin, L., & De Bortoli, M. (2008). Psychometric and comparative study of an argentine version of the morningness composite and the early/late preference scales. *Chronobiology International,* 25(1), 133-143.

26. Goel, N., Banks, S., Lin, L., Mignot, E., & Dinges, D. F. (2011). Catechol-O-methyltransferase Val158Met polymorphism associates with individual differences in sleep physiologic responses to chronic sleep loss. *PLoS One*, 6(12), 27.

27. Gomes, A. A., Tavares, J., Azevedo, M.H.P de. (2011). Sleep an academic performance in undergraduates: A multi-measure, multi-predictor approach. *Chronobiology International,* 28, 786-801.

28. Greenwood, K. M. (1994). Long-term stability and psychometric properties of the Composite Scale of Morningness. *Ergonomics*, 37, 377-383.

29. Haraszti, R. Á., Purebl, G., Salavecz, G., Poole, L., Dockray, S., & Steptoe, A. (2014). Morningness–eveningness interferes with perceived health, physical activity, diet and stress levels in working women: A cross-sectional study. *Chronobiology International, 31(7),* 829-837.

30. Horzum, M. B., Önder, İ., & Beşoluk, Ş. (2014). Chronotype and academic achievement among online learning students. *Learning and Individual Differences*, 30, 106-111.

31. Ianni, P. A., & Lafreniere, K. D. (2014). Personality and motivational correlates of energy drink consumption and misuse among female undergraduate students. *Personality and Individual Differences*, 69, 110-114.

32. Jankowski, K. S., Vollmer, C., Linke, M., & Randler, C. (2014). Differences in sun time within the same time zone affect sleep-wake and social rhythms, but not morningness preference: Findings from a Polish-German comparison study. *Time & Society*, 23, 258–276.

33. Jeong, S. H., Yu, J. C., Lee, C. H., Choi, K. S., Choi, J. E., Kim, S. H., & Joo, E. J. (2014). Human CLOCK gene-associated attention deficit hyperactivity disorder-related features in healthy adults: quantitative association study using Wender Utah Rating Scale. *European Archives of Psychiatry and Clinical Neuroscience*, 264(1), 71-81.

34. Kasof, J. (2001). Eveningness and bulimic behavior. *Personality and Individual Differences*, 31, 361-369.

35. Klei, L., Reitz, P., Miller, M., Wood, J., Maendel, S., Gross, D., Waldner, T., Eaton, J., Monk, T. H., Nimgaonkar, V. L. (1998). Heritability of morningness-eveningness and self-report sleep measures in a family based sample of 521 Hutterites. *Chronobiology International*, 22, 1041-1054.

36. Kolomeichuk, S., Randler, C., Churov, A., & Borisenkov, M. (2015). Psychometric properties of the Russian version of the Composite Scale of Morningness. *Biological Rhythm Research*, 46 (5), 725-737 --- only data from adults used.

37. Lau, E. Y. Y., Wong, M. L., Ng, E. C. W., Hui, C. C. H., Cheung, S. F., & Mok, D. S. Y. (2013). "Social Jetlag" in Morning-Type College Students Living On Campus: Implications for Physical and Psychological Well-being. *Chronobiology International, 30*(7), 910-918.

38. Lee, J. H., Kim, S. J., Lee, S. Y., Jang, K. H., Kim, I. S., & Duffy, J. F. (2014). Reliability and validity of the Korean version of Morningness–Eveningness Questionnaire in adults aged 20–39 years. *Chronobiology International*, 31(4), 479-486.

39. Mansour, H. A., Wood, J., Chowdari, K. V., Dayal, M., Thase, M. E., Kupfer, D. J., ... & Nimgaonkar, V. L. (2005). Circadian phase variation in bipolar I disorder. *Chronobiology International*, 22(3), 571-584.

40. Miller, M. A., Rothenberger, S. D., Hasler, B. P., Donofry, S. D., Wong, P. M., Manuck, S. B., ... & Roecklein, K. A. (2015). Chronotype predicts positive affect rhythms measured by ecological momentary assessment. *Chronobiology International, 32*(3), 376-384.

41. Monk, T. H., Buysse, D. J., Billy, B. D., Fletcher, M. E., Kennedy, K. S., Schlarb, J. E., & Beach, S. R. (2011). Circadian type and bed-timing regularity in 654 retired seniors: correlations with subjective sleep measures. *Sleep*, *34*(2), 235.

42. Morera-Fumero, A. L., Abreu-Gonzalez, P., Henry-Benítez, M., Díaz-Mesa, E., Yelmo-Cruz, S., & Gracia-Marco, R. (2013). Chronotype as modulator of morning serum melatonin levels. *Actas Esp Psiquiatr*, 41(3), 149-53.

43. Natale, V., & Di Milia, L. (2011). Season of birth and morningness: comparison between the northern and southern hemispheres. *Chronobiology International*, 28, 727–730.

44. Ojeda, D. A., Perea, C. S., Niño, C. L., Gutiérrez, R. M., López-León, S., Arboleda, H., ... & Forero, D. A. (2013). A novel association of two non-synonymous polymorphisms in PER2 and PER3 genes with specific diurnal preference subscales. *Neuroscience Letters*, 553, 52-56.

45. Önder, İ., Beşoluk, Ş., & Horzum, M. B. (2013). Psychometric properties of the Turkish version of the composite scale of morningness. *The Spanish Journal of Psychology*, 16, E67.

46. Park, C. I., An, S. K., Kim, H. W., Koh, M. J., Namkoong, K., Kang, J. I., & Kim, S. J. (2015). Relationships between chronotypes and affective temperaments in healthy young adults. *Journal of Affective Disorders*, 175, 256-259.

47. Pica, G., Amato, C., Pierro, A., & Kruglanski, A. W. (2015). The early bird gets the worm: On locomotors' preference for morningness. *Personality and Individual Differences*, 76, 158-160.

48. Pornpitakpan, C. (1998). Psychometric properties of the composite scale of morningness: a shortened version. *Personality and Individual Differences*, 25, 699-709.

49. Pornpitakpan, C. (2000). Additional validity of the Basic Language Moringness (BALM) Scale. *Personality and Individual Differences,* 28, 59-72.

50. Prat, G., & Adan, A. (2013). Relationships among circadian typology, psychological symptoms, and sensation seeking. *Chronobiology International, 30*(7), 942-949.

51. Randler, C. (2015). Germany, unpublished data.

52. Randler, C., Horzum, M. B., & Vollmer, C. (2014). Internet addiction and its relationship to chronotype and personality in a Turkish university student sample. *Social Science Computer Review,* 32, 484-495.

53. Randler, C., Prokop, P., Sahu, S., & Haldar, P. (2015). Cross-cultural comparison of seven morningness and sleep–wake measures from Germany, India and Slovakia. *International Journal of Psychology*, 50, 279-287.

54. Roecklein, K. A., Wong, P. M., Franzen, P. L., Hasler, B. P., Wood-Vasey, W. M., Nimgaonkar, V. L., ... & Manuck, S. B. (2012). Melanopsin gene variations interact with season to predict sleep onset and chronotype. *Chronobiology international,* 29(8), 1036-1047.

55. Roeser, K., Riepl, K., Randler, C., & Kübler, A. (2015). Effects of Chronotype and Synchrony/Asynchrony on Creativity. *Journal of Individual Differences*, 36, 131-137.

56. Sánchez López, M. P., & Díaz Morales, J. F. (2001). Tipología circadiana y estilos de personalidad en mujeres universitarias argentinas. *Psicodebate*, 97-117.

57. Savis, J. C., Eliot, J. F., Gansneder, B., & Rotella, R. J. (1997). A subjective means of assessing college athletes's sleep: A modification of the morningness/eveningness questionnaire. *International Journal of Sports Psychology*, 28, 157-171.

58. Seleem, M. A., Merranko, J. A., Goldstein, T. R., Goldstein, B. I., Axelson, D. A., Brent, D. A., ... & Birmaher, B. (2015). The longitudinal course of sleep timing and circadian preferences in adults with bipolar disorder. *Bipolar disorders.* In press.

59. Smith, C. S., Folkard, S., Schmieder, R. A., Parra, L. F., Spelten, E., Almiral, H., Sen, R. N., Sahu, S., Perez, L. M., & Tisak, J. (2002). Investigation of morning-evening orientation in six countries using the preferences scale. *Personality and Individual Differences*, 32, 949-968.

60. Soehner, A. M., Kennedy, K. S., & Monk, T. H. (2007). Personality correlates with sleep-wake variables. *Chronobiology International,* 24, 889-903.

61. Soehner, A. M., Kennedy, K. S., & Monk, T. H. (2011). Circadian preference and sleep-wake regularity: associations with self-report parameters in daytime working adults. *Chronobiology International*, 28, 802-809.

62. Stolarski, M., & Jankowski, K. S. (2015). Morningness–eveningness and performance-based emotional intelligence. *Biological Rhythm Research*, 46(3), 417-423.

63. Thun, E., Bjorvatn, B., Osland, T., Steen, V. M., Sivertsen, B., Johansen, T., Lilleholt, T. H., Udnes, I., Nordhus, I. H., & Pallesen, S. (2012). An actigraphy validation study of seven morningness-eveningness inventories. *European Psychologist*, 17, 222-230.

64. Voinescu, B. I., Coogan, A. N., Thome, J., & Orasan, R. (2010). Psychometric properties of the romanian version of the composite scale of morningness in healthy adults. *Cognitie, Creier, Comportament*, 14(1), 37-46.

65. Voinescu, B., Vesa, J., & Coogan, A. (2011). Self-reported diurnal preference and sleep disturbance in type 2 diabetes mellitus. *Acta Endocrinologica-Bucharest*, 7(1), 69-82.

66. Voinescu, B. I., & Szentagotai, A. (2015). Sleep hygiene awareness: its relation to sleep quality and diurnal preference. *Journal of Molecular Psychiatry*, 3, 1.

67. Waage, S., Moen, B. E., Pallesen, S., Eriksen, H. R., & Ursin, H. (2009). Shift work disorder among oil rig workers in the North Sea. *Sleep*, 32(4), 558. --- day shift used

68. Wood, J., Birmaher, B., Axelson, D., Ehmann, M., Kalas, C., Monk, K., ... & Nimgainkar, V. L. (2009). Replicable differences in preferred circadian phase between bipolar disorder patients and control individuals. *Psychiatry Research*, 166(2), 201-209.

69. Zickar, M. J., Russell, S. S., Smith, C. S., Bohle, P., & Tilley, A. J. (2002). Evaluating two morningness scales with item response theory. *Personality and Individual Differences*, 33(1), 11-24.

Supplement 2: Database used for the study. Comments column: information is given, when subsamples have been used (e.g. healthy controls) or when data on temperature/climate have been collected from a climate station nearby. Data on temperature and photoperiod have been taken from different sources and places, when a nearby place was used, the information is given. Please note that the supplemental data are also uploaded by SPSS file (sav) and Excel.

No	author	year	country	location	comment	mean_age	age_SD	total_sample	CSM_mean	SD_CSM	percent_female	Latitude_mirror	mean_temp	source_temp	sunrise	photoperiod_hh_min	sunset	comment_sunrise
1	Adan et al.	2010	Spain	Barcelona		21,98	2,86	850	30,74	7,29	53,41	41,00	15,50	klimadiagramme.de	06:18:00	15:10	21:28:00	
2	Ahn et al.	2008	SouthKorea	Seoul	only control group used	33,70	7,90	95	32,60	5,70	48,42	37,00	11,80	klimadiagramme.de	05:11:00	14:45	19:56:00	
3	Arbabi, Tayebe	2009	Iran	Mashhad		21,21	1,05	499	32,72	6,79	48,50	36,00	13,60	klimadiagramme.de	05:14:00	14:38	19:52:00	
4	Bae	2014	SouthKorea	Seoul		33,50	9,20	313	35,25	6,80	61,02	37,00	11,80	klimadiagramme.de	05:11:00	14:45	19:56:00	
5	Bhatia	2015	India	Delhi		21,05	1,67	83	43,12	4,13	100,00	28,00	25,00	klimadiagramme.de	05:24:00	13:58	19:22:00	
5	Bhatia	2015	India	Delhi		41,50	11,93	160	43,43	5,17	74,38	28,00	25,00	klimadiagramme.de	05:24:00	13:58	19:22:00	
5	Bhatia	2015	India	Delhi		30,97	14,60	150	42,94	5,78	32,67	28,00	25,00	klimadiagramme.de	05:24:00	13:58	19:22:00	
6	Blagrove & Akehurst	2000	UK	Wales		21,30	3,50	93	34,69	6,76	49,46	51,00	11,00	weatherbase.com	04:58:00	16:39	21:37:00	Wales
7	Bohle et al.	2001	Australia	Brisbane/Sydney		22,50		670	32,90	7,50	64,26	30,00	20,60	klimadiagramme.de	04:50:00	13:52	18:42:00	Brisbane
8	Boudebesse et al.	2013	France	Créteil	healthy control; PARIS used for temperature	42,04	11,50	156	39,64	7,90	53,85	48,00	10,60	klimadiagramme.de	05:47:00	16:10	21:57:00	Paris
9	Brown	1993	USA	Pennsylvania	HARRISBURG used for temperature	39,54	9,84	148	41,04		20,27	41,00	11,80	weatherbase.com	05:38:00	15:02	20:40:00	Harrisburg
10	Caci et al.	1999	France	Nice		25,60	6,10	356	37,31	6,36	85,11	43,00	15,30	klimadiagramme.de	05:49:00	15:26	21:15:00	
11	Caci et al.	2000	France	Nice		22,51	4,37	60	38,33	6,91	81,67	43,00	15,30	klimadiagramme.de	05:49:00	15:26	21:15:00	
12	Caci et al.	2005	Thai	Bangkok		17,64	1,25	503	34,34	5,59	74,55	14,00	28,40	klimadiagramme.de	05:52:00	12:56	18:48:00	
12	Caci et al.	2005	Italy	Bologna		23,01	3,94	702	32,73	6,25	60,26	44,00	13,21	klimadiagramme.de	05:30:00	15:33	21:03:00	
12	Caci et al.	2005	France	Nice		24,29	5,40	627	36,80	6,43	67,62	43,00	15,30	klimadiagramme.de	05:49:00	15:26	21:15:00	
12	Caci et al.	2005	France	Nice		23,00	5,24	552	36,57	6,55	64,86	43,00	15,30	klimadiagramme.de	05:49:00	15:26	21:15:00	
12	Caci et al.	2005	Australia	Sydney		22,45	6,89	654	33,10	7,53	63,61	33,00	17,90	klimadiagramme.de	05:41:00	14:24	20:05:00	
12	Caci et al.	2005	Spain	Barcelona		19,94	2,22	391	30,74	6,54	66,24	41,00	15,50	klimadiagramme.de	06:18:00	15:10	21:28:00	
13	Caci et al.	2009	France	Nice		23,00	4,48	442	33,35	6,77	64,48	43,00	15,30	klimadiagramme.de	05:49:00	15:26	21:15:00	
14	Di Milia & Mueller	2012	Australia	Rockhampton		36,28	12,94	184	39,93	8,80	44,57	23,00	22,44	climate-charts.com	05:09:00	13:34	18:43:00	
15	Diaz-Morales & Sanchez-Lopez	2005	Peru	Lima		21,73	2,63	139	33,88	6,24	78,40	12,00	19,20	klimadiagramme.de	05:41:00	12:50	18:31:00	

16	Diaz-Morales & Sanchez-Lopez	2004	Spain	Madrid		21,27	1,60	203	28,90	6,14	81,80	40,00	15,30	klimadiagramme.de	06:45:00	15:03	21:48:00	
16	Diaz-Morales & Sanchez-Lopez	2004	Spain	Madrid		49,10	6,31	125	35,57	5,55	66,70	40,00	14,40	klimadiagramme.de	06:45:00	15:03	21:48:00	
17	Diaz-Morales	2007	Spain	Madrid	PAID, personality styles	21,53	2,77	360	30,55	6,93	76,39	40,00	15,30	klimadiagramme.de	06:45:00	15:03	21:48:00	
18	Diaz-Morales & Sanchez-Lopez	2008	Spain	Madrid	PAID sex/gender	50,06	6,40	559	38,20	6,95	41,68	40,00	15,30	klimadiagramme.de	06:45:00	15:03	21:48:00	
19	Diaz-Morales & Aparicio	2003	Spain	Madrid		29,05	12,56	189	31,88	7,37	67,72	40,00	15,30	klimadiagramme.de	06:45:00	15:03	21:48:00	
20	Diaz Ramiro	2000	Spain	Madrid	cited in Diaz.Morales Psicothema 2005	20,34		314	31,07	6,40	81,00	40,00	15,30	klimadiagramme.de	06:45:00	15:03	21:48:00	
21	Digdon	2010	Canada	Edmonton		21,21	5,50	461	31,99	7,09	71,14	53,00	3,50	klimadiagramme.de	05:05:00	17:02	22:07:00	
22	Digdon & Landry	2013	Canada	Edmonton		21,80	6,80	219	31,86	6,47	71,69	53,00	3,50	klimadiagramme.de	05:05:00	17:02	22:07:00	
23	Dresch et al.	2005	Brasil	Novo Hamburgo	Porto Alegre used for temperature	24,20	7,20	804	35,26	7,32	62,06	29,00	19,50	klimadiagramme.de	06:21:00	14:05	20:26:00	Porto Alegre
24	Driscoll et al.	2008	USA	Pittsburgh		78,90	3,30	64	44,90	5,40	46,90	40,00	10,20	klimadiagramme.de	05:50:00	15:03	20:53:00	
25	Gil et al.	2008	Argentina	La Pampa	San Luis, Mendoza, General Pico // La Pampa used for geography	33,64	14,00	304	36,41	7,65	69,10	37,00	16,70	klimadiagramme.de	06:24:00	14:19	20:43:00	Mendoza
26	Goel et al.	2011	USA	Pennsylvania	Penn State college	29,97		129	39,88		48,84	41,00	9,00	weatherbase.com	05:32:00	15:00	20:32:00	Pennsylvania
27	Gomes et al.	2011	Portugal	Coimbra/Aveiro		19,98	1,65	1.654	32,20	5,76	55,00	40,00	15,00	weatherbase.com	06:01:00	15:09	21:10:00	
28	Greenwood	1994	Australia	La Trobe		21,30		424	36,10	6,70	88,10	38,00	14,60	klimadiagramme.de	05:55:00	14:47	20:42:00	Melbourne
29	Haraszti et al.	2014	Hungary	Budapest		37,56	10,68	202	37,19	7,59	100,00	47,00	10,40	klimadiagramme.de	04:47:00	15:58	20:45:00	
30	Horzum et al.	2014	Turkey	Ankara		25,63	6,63	724	35,40	6,10	49,86	39,00	11,71	klimadiagramme.de	05:20:00	15:00	20:20:00	
31	Ianni & Lafreniere	2014	Canada	Ontario		20,90	2,40	96	30,74	7,35	100,00	42,00	9,90	weatherbase.com	05:55:00	15:16	21:11:00	
32	Jankowski et al.	2014	Poland	Warsaw		20,81	1,73	291	32,37	6,72	82,82	52,00	7,80	klimadiagramme.de	04:14:00	16:46	21:00:00	
33	Jeong et al.	2014	SouthKorea	Seoul		25,06	6,05	1.289	30,67	6,00	63,07	37,00	11,80	klimadiagramme.de	05:11:00	14:45	19:56:00	
34	Kasof	2001	USA	California		22,70	4,90	151	38,60	6,11	62,25	34,00	17,20	selbst berechnet.com	05:41:00	14:23	20:04:00	
35	Klei et al.	2005	Canada	Manitoba	WINNIPEG used for temperature	37,30		521	41,00	5,80	65,26	50,00	2,40	climatecharts.com	05:20:00	16:21	21:41:00	Winnipeg
36	Kolomeichuck et al.	2015	Russia	Petrozavodsk	adults only	21,74	2,96	298	31,58	6,33	64,43	61,78	3,20	klimadiagramme.de	03:35:00	18:50	22:25:00	St. Petersburg
37	Lau et al.	2013	China/Hongkong	HongKong	Hongkong/Macau/ Hongkong Observatory for climate data	20,20	2,30	1.263	31,38	5,93	66,50	22,00	22,93	klimadiagramme.de	05:40:00	13:30	19:10:00	
38	Lee et al.	2014	SouthKorea	Seoul		22,90	2,14	299	31,90	6,39	36,12	37,00	11,80	klimadiagramme.de	05:11:00	14:45	19:56:00	

39	Mansour et al.	2005	USA	Pittsburgh	only control group used	33,00	10,10	349	37,30	7,80	52,15	40,00	10,20	klimadiagramme.de	05:50:00	15:03	20:53:00	
40	Miller et al.	2015	USA	Pittsburgh		42,89	7,33	408	39,52	7,13	51,70	40,00	10,20	klimadiagramme.de	05:50:00	15:03	20:53:00	
41	Monk et al.	2011	USA	Pittsburgh area		74,70	6,00	654	41,30	6,60	44,50	40,00	10,20	klimadiagramme.de	05:50:00	15:03	20:53:00	
42	Morera-Fumero et al.	2013	Tenerife	La Laguna	Santa Cruz de Tenerife used for temperature	41,50	11,40	118	36,90	6,90	49,00	28,00	21,20	klimadiagramme.de	07:08:00	13:57	21:05:00	Santa Cruz
43	Natale & Di Milia	2011	Italy	Bologna		23,60	4,35	933	32,50	6,43	59,16	44,00	13,21	klimadiagramme.de	05:30:00	15:33	21:03:00	
43	Natale & Di Milia	2011	Australia	Rockhampton		24,79	7,45	732	36,70	7,37	68,12	23,00	22,44	climate-charts.com	05:09:00	13:34	18:43:00	
44	Ojeda et al.	2013	Colombia	Bogota		20,80	2,70	209	39,97	5,63	70,33	4,00	13,30	klimadiagramme.de	05:47:00	12:23	18:10:00	
45	Önder et al.	2013	Turkey	Sakarya		22,58	3,13	650	34,38	6,17	52,60	41,00	14,12	klimadiagramme.de	05:32:00	15:07	20:39:00	Istanbul
46	Park et al.	2015	SouthKorea	Seoul		22,70	3,71	641	32,85	6,00	41,34	37,00	11,80	klimadiagramme.de	05:11:00	14:45	19:56:00	
47	Pica et al.	2015	Italy	Rome		24,69	5,08	342	32,19	5,94	67,54	41,00	15,40	klimadiagramme.de	05:35:00	15:13	20:48:00	
48	Pornpitakpan	1998	Thai	Bangkok		18,26	1,04	321	35,30	6,10	90,34	14,00	28,40	klimadiagramme.de	05:52:00	12:56	18:48:00	
49	Pornpitakpan	2000	Singapore	Singapore		20,68	1,15	100	32,37	5,84	79,00	1,00	27,30	klimadiagramme.de	07:01:00	12:11	19:12:00	
50	Prat & Adan	2013	Spain	Barcelona		21,40	2,90	517	30,32	7,13	66,54	41,00	15,50	klimadiagramme.de	06:18:00	15:10	21:28:00	
51	Randler	2015	Germany	SW Germany	KARLSRUHE for temperature	18,99	0,83	1.812	32,89	6,30	71,30	49,00	10,30	klimadiagramme.de	05:19:00	16:17	21:36:00	Mannheim
51	Randler	2015	Germany	SW Germany	KARLSRUHE for temperature	23,76	2,37	3.340	33,22	6,37	62,50	49,00	10,30	klimadiagramme.de	05:19:00	16:17	21:36:00	Mannheim
51	Randler	2015	Germany	SW Germany	KARLSRUHE for temperature	57,98	6,13	356	39,30	6,31	74,70	49,00	10,30	klimadiagramme.de	05:19:00	16:17	21:36:00	Mannheim
51	Randler	2015	Germany	SW Germany	KARLSRUHE for temperature	40,25	6,13	734	37,04	7,17	75,30	49,00	10,30	klimadiagramme.de	05:19:00	16:17	21:36:00	Mannheim
52	Randler et al.	2014	Turkey	Sakarya	ISTANBUL used for temperature	20,81	1,97	616	34,11	6,41	72,10	41,00	14,12	klimadiagramme.de	05:32:00	15:07	20:39:00	Istanbul
53	Randler et al.	2015	India	Kalyani	Calcutta used for temperature	20,02	1,23	409	37,10	6,32	53,79	23,00	26,90	klimadiagramme.de	04:53:00	13:31	18:24:00	Calcutta
53	Randler et al.	2015	Slovakia	Trnava	PIESTANY used for temperature	20,56	4,27	482	33,43	6,00	81,95	48,00	9,00	weatherbase.com	04:51:00	16:04	20:55:00	Bratislava
54	Roecklein et al.	2012	USA	Pittsburgh		41,90	7,70	233	39,35	7,13	46,60	40,00	10,20	klimadiagramme.de	05:50:00	15:03	20:53:00	
55	Roeser et al.	2015	Germany	Würzburg	GEISENHEIM used for temperature	22,82	5,83	153	32,95	5,67	83,40	50,00	9,40	klimadiagramme.de	05:12:00	16:20	21:32:00	
56	Sanchez-Lopez & Diaz-Morales	2001	Argentina	oeste del Argentina	cited in Diaz.Morales Psicothema 2005; CORDOBA used for temperature	21,02	1,80	120	34,30	5,16	100,00	31,00	17,30	klimadiagramme.de	06:09:00	14:11	20:20:00	Cordoba
57	Savis et al.	1997	USA	Charlottesville VA		19,96		65	33,86	5,88	46,15	38,00	14,00	weatherbase.com	05:52:00	14:48	20:40:00	
58	Seleem et al.	2015	USA	Pittsburgh	healthy controls used	40,80	7,30	55	40,50	6,50	21,80	40,00	10,20	klimadiagramme.de	05:50:00	15:03	20:53:00	

59	Smith et al.	2002	UK	SwanseaSheffield	Swansea & Sheffield: means used	20,30		165	31,60	6,30	60,70	52,00	9,80	klimadiagramme.de	04:58:00	16:39	21:37:00	Wales
59	Smith et al.	2002	Netherlands	Maastricht		21,10		281	30,10	7,40	73,10	51,00	9,50	klimadiagramme.de	05:24:00	16:31	21:55:00	
59	Smith et al.	2002	Spain	Barcelona		19,90		159	33,90	5,80	79,20	41,00	15,50	klimadiagramme.de	06:18:00	15:10	21:28:00	
59	Smith et al.	2002	Colombia	Bogota	BOGOTA used for temperature	23,60		293	42,40	5,70	65,30	5,00	13,30	klimadiagramme.de	05:47:00	12:23	18:10:00	
59	Smith et al.	2002	USA	Ohio		19,00		551	31,40	6,80	70,50	41,00	10,10	weatherbase.com	05:27:00	14:42	20:09:00	
59	Smith et al.	2002	India	Calcutta		21,80		300	39,40	6,40	39,70	22,00	26,90	klimadiagramme.de	04:53:00	13:31	18:24:00	
60	Soehner et al.	2007	USA	Pittsburgh		32,40	9,90	54	36,60	7,50	44,30	40,00	10,20	klimadiagramme.de	05:50:00	15:03	20:53:00	
61	Soehner et al.	2011	USA	Pittsburgh		31,51	5,93	62	37,15	7,60	66,10	40,00	10,20	klimadiagramme.de	05:50:00	15:03	20:53:00	
62	Stolarski & Jankowski	2014	Poland	Warsaw		21,60	2,62	182	32,85	7,18	73,63	52,00	7,80	klimadiagramme.de	04:14:00	16:46	21:00:00	
63	Thun et al.	2013	Norway	Bergen		21,40	2,20	166	33,20	6,40	56,02	60,00	7,80	klimadiagramme.de	04:10:00	19:01	23:11:00	
64	Voinescu et al.	2010	Romania	Cluj-Napoca		35,14	14,45	205	37,36	7,24	65,40	46,00	8,20	klimadiagramme.de	05:32:00	15:51	21:23:00	
65	Voinescu et al.	2011	Romania	Cluj-Napoca	only control group used	47,16	10,53	102	42,00		51,96	46,00	8,20	klimadiagramme.de	05:32:00	15:51	21:23:00	
66	Voinescu & Szentagotai	2015	Romania	Cluj-Napoca used	three large cities in Romania	29,20	10,90	652	34,80	7,40	73,60	46,00	8,20	klimadiagramme.de	05:32:00	15:51	21:23:00	
67	Waage et al.	2009	Norway	Bergen used	geographical mean from Norway used: 64°N, 5% women used	39,80	10,20	96	37,72	4,95	5,00	64,00	7,80	klimadiagramme.de	04:10:00	19:01	23:11:00	
68	Wood et al.	2009	USA	Pittsburgh	only control group from study; MEDIAN used	40,51		128	39,00		76,60	40,00	10,20	klimadiagramme.de	05:50:00	15:03	20:53:00	
69	Zickar et al.	2002	USA	Bowling Green		42,50		180	36,86	9,07		41,00	10,10	waetherbase.com	05:27:00	14:42	20:09:00	

Supplement3: Excluded studies and exclusion criteria.

PT J --- excluded adolescents
AU Randler, C
Weber, V
AF Randler, Christoph
Weber, Vanessa
TI Positive and negative affect during the school day and its relationship to morningness-eveningness
SO BIOLOGICAL RHYTHM RESEARCH

PT J --- excluded review
AU Tonetti, L
   Adan, A
   Di Milia, L
   Randler, C
   Natale, V
AF Tonetti, L.
   Adan, A.
   Di Milia, L.
   Randler, C.
   Natale, V.
TI Measures of circadian preference in childhood and adolescence: A review
SO EUROPEAN PSYCHIATRY
AB Purpose:

PT J --- excluded Randler study, explanation see methods
AU Randler, C
Luffer, M
Muller, M
AF Randler, Christoph
Luffer, Manuela
Mueller, Melanie
TI Morningness in Teachers is Related to a Higher Sense of Coherence and
Lower Burnout
SO SOCIAL INDICATORS RESEARCH
TC 0
Z9 0
SN 0303-8300
EI 1573-0921
PD JUN
PY 2015
VL 122
IS 2
BP 595
EP 606
DI 10.1007/s11205-014-0699-2
UT WOS:000353792100014
ER

PT J --- internet sampling across USA (whole country)
AU Wang, LL
   Chartrand, TL
AF Wang, Lili
   Chartrand, Tanya L.
TI Morningness-Eveningness and Risk Taking
SO JOURNAL OF PSYCHOLOGY
TC 0
Z9 0
SN 0022-3980
EI 1940-1019
PD MAY 19
PY 2015
VL 149
IS 4
BP 394
EP 411
DI 10.1080/00223980.2014.885874
UT WOS:000353335000005
PM 25901637
ER

PT J --- excluded children
AU Arbabi, T
Vollmer, C
Dorfler, T
Randler, C
AF Arbabi, Talat
Vollmer, Christian
Doerfler, Tobias
Randler, Christoph
TI The influence of chronotype and intelligence on academic achievement in primary school is mediated by conscientiousness, midpoint of sleep and motivation
SO CHRONOBIOLOGY INTERNATIONAL
TC 0
Z9 0
SN 0742-0528
EI 1525-6073
PD APR
PY 2015
VL 32
IS 3
BP 349
EP 357

PT J --- CSM scores not reported
AU Kang, JI
Park, CI
Sohn, SY
Kim, HW
Namkoong, K
Kim, SJ
AF Kang, Jee In
Park, Chun Il
Sohn, Sung-yun
Kim, Hae Won
Namkoong, Kee
Kim, Se Joo
TI Circadian preference and trait impulsivity, sensation-seeking and response inhibition in healthy young adults
SO CHRONOBIOLOGY INTERNATIONAL
TC 0
Z9 0
SN 0742-0528
EI 1525-6073
PD MAR
PY 2015
VL 32
IS 2
BP 235
EP 241
DI 10.3109/07420528.2014.965313
UT WOS:000351005100008

PT J --- excluded Randler study, explanation see methods
AU Randler, C
Goma-i-Freixanet, M
Muro, A
Knauber, C
Adan, A
AF Randler, Christoph
Goma-i-Freixanet, Montserrat
Muro, Anna
Knauber, Christina
Adan, Ana
TI Do different circadian typology measures modulate their relationship
with personality? A test using the Alternative Five Factor Model
SO CHRONOBIOLOGY INTERNATIONAL

TC 0
Z9 0
SN 0742-0528
EI 1525-6073
PD 00351005100015
PM 25290037
ER

PT J --- excluded Randler, explanation see methods
AU Werner, L
Geisler, J
Randler, C
AF Werner, Laura
Geisler, Jennifer
Randler, Christoph
TI Morningness as a Personality Predictor of Punctuality
SO CURRENT PSYCHOLOGY
TC 0
Z9 0
SN 1046-1310
EI 1936-4733
PD MAR
PY 2015
VL 34
IS 1
BP 130
EP 139
DI 10.1007/s12144-014-9246-1
UT WOS:000350802900011
ER

PT J ---excluded whole USA
AU Cole, J
AF Cole, James S.
TI A survey of college-bound high school graduates regarding circadian
preference, caffeine use, and academic performance
SO SLEEP AND BREATHING
TC 0
Z9 0
SN 1520-9512
EI 1522-1709
PD MAR
PY 2015
VL 19
IS 1
BP 123
EP 127
DI 10.1007/s11325-014-0976-y
UT WOS:000349983900027
PM 24788599
ER

PT J --- not CSM

AU Simor, P
   Zavecz, Z
   Palosi, V
   Torok, C
   Koteles, F
AF Simor, Peter
   Zavecz, Zsofia
   Palosi, Vivien
   Toeroek, Csenge
   Koeteles, Ferenc
TI The influence of sleep complaints on the association between chronotype
   and negative emotionality in young adults
SO CHRONOBIOLOGY INTERNATIONAL
TC 0
Z9 0
SN 0742-0528
EI 1525-6073
PD FEB

PT J --- clinical
AU Lee, YJ
Park, J
Kim, S
Cho, SJ
Kim, SJ
AF Lee, Yu Jin
Park, Juhyun
Kim, Soohyun
Cho, Seong-Jin
Kim, Seog Ju
TI Academic Performance among Adolescents with Behaviorally Induced
Insufficient Sleep Syndrome
SO JOURNAL OF CLINICAL SLEEP MEDICINE
TC 0
Z9 0
SN 1550-9389
EI 1550-9397
PY 2015
VL 11
IS 1
BP 61
EP 68
DI 10.5664/jcsm.4368
UT WOS:000351167800011
PM 25515277
ER

PT J – no CSM data
AU Putilov, AA
Donskaya, OG
Verevkin, EG
AF Putilov, Arcady A.
Donskaya, Olga G.
Verevkin, Evgeniy G.
TI How many diurnal types are there? A search for two further "bird species"
SO PERSONALITY AND INDIVIDUAL DIFFERENCES
TC 0
Z9 0
SN 0191-8869
PD JAN
PY 2015
VL 72
BP 12
EP 17
DI 10.1016/j.paid.2014.08.003
UT WOS:000351193400003
ER

PT J --- overlap with Jankowski et al. (2014)
AU Jankowski, KS
AF Jankowski, K. S.
TI Composite Scale of Morningness: Psychometric properties, validity with
   Munich ChronoType Questionnaire and age/sex differences in Poland
SO EUROPEAN PSYCHIATRY
TC 7
Z9 7
SN 0924-9338
EI 1778-3585
PD JAN
PY 2015
VL 30
IS 1
DI 10.1016/j.eurpsy.2014.01.004
UT WOS:000348032300026
PM 24630377
ER

PT J --- adolescents
AU Randler, C
Rahafar, A
Arbabi, T
Bretschneider, R
AF Randler, Christoph
Rahafar, Arash
Arbabi, Talat
Bretschneider, Rebekka
TI Affective State of School Pupils During Their First Lesson of the
Day-Effect of Morningness-Eveningness
SO MIND BRAIN AND EDUCATION
TC 1
Z9 1
SN 1751-2271
EI 1751-228X
PD DEC
PY 2014
VL 8
IS 4
BP 214
EP 219
DI 10.1111/mbe.12060
UT WOS:000345227900006
ER

PT J --- adolescents

AU Randler, C
   Vollmer, C
   Besoluk, S
   Onder, I
   Horzum, MB
AF Randler, Christoph
   Vollmer, Christian
   Besoluk, Senol
   Oender, Ismail
   Horzum, Mehmet Baris
TI Age and gender differences in morningness-eveningness in Turkish
   adolescents and young adults
SO BIOLOGICAL RHYTHM RESEARCH

TC 1
Z9 1
SN 0929-1016
EI 1744-4179
PD MAR 4
PY 2014
VL 45
IS 2
BP 277
EP 284
DI 10.1080/09291016.2013.805915
UT WOS:000332277000013
ER

PT J CSM scores not reported
AU Bhatti, P
   Mirick, DK
   Davis, S
AF Bhatti, Parveen
   Mirick, Dana K.
   Davis, Scott
TI The impact of chronotype on melatonin levels among shift workers
SO OCCUPATIONAL AND ENVIRONMENTAL MEDICINE
TC 5
Z9 5
SN 1351-0711
EI 1470-7926
PD MAR
PY 2014
VL 71
IS 3
BP 195
EP 200
DI 10.1136/oemed-2013-101730
UT WOS:000330849700008
PM 24399070
ER

PT J – no CSM
AU Biswas, A
Adan, A
Haldar, P
Majumder, D
Natale, V
Randler, C
Tonetti, L
Sahu, S
AF Biswas, Animesh
Adan, Ana
Haldar, Prasun
Majumder, Debasish
Natale, Vincenzo
Randler, Christoph
Tonetti, Lorenzo
Sahu, Subhashis
TI Exploration of transcultural properties of the reduced version of the Morningness-Eveningness Questionnaire (rMEQ) using adaptive neuro-fuzzy inference system
SO BIOLOGICAL RHYTHM RESEARCH
TC 1
Z9 1
SN 0929-1016
EI 1744-4179
PY 2014
VL 45
IS 6
BP 955
EP 968
DI 10.1080/09291016.2014.939442
UT WOS:000342312800012
ER

PT J --- duplicate dataset Jankowski et al. (2014)
AU Randler, C
Jankowski, KS
AF Randler, Christoph
Jankowski, Konrad S.
TI Evidence for the validity of the composite scale of morningness based on students from Germany and Poland - relationship with sleep-wake and social schedules
SO BIOLOGICAL RHYTHM RESEARCH
TC 2
Z9 2
SN 0929-1016
EI 1744-4179
PY 2014
VL 45
IS 4
BP 653
EP 659
DI 10.1080/09291016.2014.884307
UT WOS:000337580100017
ER

PT J --- overlap with Monk et al. (2011)
AU Monk, TH
Buysse, DJ
AF Monk, Timothy H.
Buysse, Daniel J.
TI Chronotype, bed timing and total sleep time in seniors
SO CHRONOBIOLOGY INTERNATIONAL
TC 1
Z9 1
SN 0742-0528
EI 1525-6073
PY 2014
VL 31
IS 5
BP 655
EP 659
DI 10.3109/07420528.2014.885981
UT WOS:000336971900006
PM 24517139
ER

PT J – clinical sample
AU Corruble, E
Frank, E
Gressier, F
Courtet, P
Bayle, F
Llorca, PM
Vaiva, G
Gorwood, P
AF Corruble, Emmanuelle
Frank, Ellen
Gressier, Florence
Courtet, Philippe
Bayle, Frank
Llorca, Pierre-Michel
Vaiva, Guillaume
Gorwood, Philip
TI Morningness-eveningness and treatment response in major depressive disorder
SO CHRONOBIOLOGY INTERNATIONAL
TC 2
Z9 2
SN 0742-0528
EI 1525-6073
PY 2014
VL 31
IS 2
BP 283
EP 289
DI 10.3109/07420528.2013.834924
UT WOS:000331148700016
PM 24128195
ER

PT J --- adolescents
AU Koscec, A
Radosevic-Vidacek, B
Bakotic, M
AF Koscec, Adrijana
Radosevic-Vidacek, Biserka
Bakotic, Marija
TI Morningness-eveningness and sleep patterns of adolescents attending school in two rotating shifts
SO CHRONOBIOLOGY INTERNATIONAL
RI Radosevic-Vidacek, Biserka/C-2986-2013; Bakotic, Marija/G-8274-2015
OI Radosevic-Vidacek, Biserka/0000-0001-7445-7335;
TC 3
Z9 3
SN 0742-0528
EI 1525-6073PM 24131149
ER

PT J ---overlap with Ojeda et al. (2013)
AU Ojeda, DA
Perea, CS
Suarez, A
Nino, CL
Gutierrez, RM
Lopez-Leon, S
Adan, A
Arboleda, H
Camargo, A
Forero, DA
AF Ojeda, Diego A.
Perea, Claudia S.
Suarez, Annjy
Nino, Carmen L.
Gutierrez, Rafael M.
Lopez-Leon, Sandra
Adan, Ana
Arboleda, Humberto
Camargo, Andres
Forero, Diego A.
TI Common functional polymorphisms in SLC6A4 and COMT genes are associated
with circadian phenotypes in a South American sample
SO NEUROLOGICAL SCIENCES
RI Adan, Ana /B-3643-2011; Forero, Diego/A-9305-2008
OI Adan, Ana /0000-0002-3328-3452; Forero, Diego/0000-0001-9175-3363

PT J children
AU Randler, C
   Truc, Y
AF Randler, Christoph
   Truc, Yvonne
TI Adaptation of the Composite Scale of Morningness for Parent Report and
   Results from Kindergarten Children
SO SWISS JOURNAL OF PSYCHOLOGY
TC 4
Z9 4
SN 1421-0185
EI 1662-0879
PY 2014
VL 73
IS 1
BP 35
EP 39
DI 10.1024/1421-0185/a000121
UT WOS:000328958700004
ER

PT J --- Randler study, explanation see methods
AU Randler, C
   Vollmer, C
AF Randler, Christoph
   Vollmer, Christian
TI AGGRESSION IN YOUNG ADULTS - A MATTER OF SHORT SLEEP AND SOCIAL JETLAG?
SO PSYCHOLOGICAL REPORTS
RI Vollmer, Christian/K-8253-2012
OI Vollmer, Christian/0000-0002-7763-7013
TC 3
Z9 3
SN 0033-2941
PD DEC
PY 2013
VL 113
IS 3
BP 754
EP 765
DI 10.2466/16.02.PR0.113x31z7
UT WOS:000331072500004
PM 24693810
ER

PT J --- sample (veterans)
AU Hasler, BP
Insana, SP
James, JA
Germain, A
AF Hasler, Brant P.
Insana, Salvatore P.
James, Jeffrey A.
Germain, Anne
TI Evening-type military veterans report worse lifetime posttraumatic stress symptoms and greater brainstem activity across wakefulness and REM sleep
SO BIOLOGICAL PSYCHOLOGY.
TC 1
Z9 1
SN 0301-0511
EI 1873-6246
PD OCT
PY 2013
VL 94
IS 2
BP 255
EP 262
DI 10.1016/j.biopsycho.2013.06.007
UT WOS:000325462900004
PM 23831547
ER

J

AU Randler, C --- Randler sample, explanation see methods

AF Randler, Christoph
TI German version of the reduced Morningness-Eveningness Questionnaire
(rMEQ)
SO BIOLOGICAL RHYTHM RESEARCH
TC 5
Z9 5
SN 0929-1016
EI 1744-4179
PD OCT 1
PY 2013
VL 44
IS 5
BP 730
EP 736
DI 10.1080/09291016.2012.739930
UT WOS:000324515500005
ER

PT J --- adolescents

AU Kauderer, S
   Randler, C
AF Kauderer, Simone
   Randler, Christoph
TI Differences in time use among chronotypes in adolescents
SO BIOLOGICAL RHYTHM RESEARCH
TC 2
Z9 2
SN 0929-1016
PD AUG 1
PY 2013
VL 44
IS 4
BP 601
EP 608
DI 10.1080/09291016.2012.721687
UT WOS:000321691400008
ER

PT J no CSM data
AU Jankowski, KS
AF Jankowski, Konrad S.
TI Polish version of the reduced MorningnessEveningness Questionnaire
SO BIOLOGICAL RHYTHM RESEARCH
TC 8
Z9 8
SN 0929-1016
PD JUN 1
PY 2013
VL 44
IS 3
BP 427
EP 433
DI 10.1080/09291016.2012.704791
UT WOS:000318779100009
ER

PT J no CSM data
AU Putilov, AA
Verevkin, EG
Donskaya, OG
AF Putilov, Arcady A.
Verevkin, Evgeniy G.
Donskaya, Olga G.
TI Overall and specific relationships between inter-individual variations in personality and sleepwake adaptability
SO BIOLOGICAL RHYTHM RESEARCH
RI Putilov, Arcady/E-2010-2011
OI Putilov, Arcady/0000-0003-2779-9046
TC 0
Z9 0
SN 0929-1016
PD APR 1
PY 2013
VL 44
IS 2
BP 287
EP 311
DI 10.1080/09291016.2012.681847
UT WOS:000315567800010
ER

PT J children
AU Onder, I
Besoluk, S
AF Onder, Ismail
Besoluk, Senol
TI Adaptation of the morningness eveningness scale for children into Turkish
SO BIOLOGICAL RHYTHM RESEARCH.
TC 1
Z9 1
SN 0929-1016
PD APR 1
PY 2013
VL 44
IS 2
BP 313
EP 323
DI 10.1080/09291016.2012.681848
UT WOS:000315567800011
ER

PT J Review
AU Di Milia, L
Adan, A
Natale, V
Randler, C
AF Di Milia, Lee
Adan, Ana
Natale, Vincenzo
Randler, Christoph
TI Reviewing the Psychometric Properties of Contemporary Circadian Typology
Measures
SO CHRONOBIOLOGY INTERNATIONAL
RI Adan, Ana /B-3643-2011
OI Adan, Ana /0000-0002-3328-3452
TC 26
Z9 26
SN 0742-0528
EI 1525-
UT WOS:000326987500008
PM 24001393
ER

PT J **clinical**, small sample
AU Boudebesse, C
Geoffroy, PA
Bellivier, F
Henry, C
Folkard, S
Leboyer, M
Etain, B
AF Boudebesse, Carole
Geoffroy, Pierre Alexis
Bellivier, Frank
Henry, Chantal
Folkard, Simon
Leboyer, Marion
Etain, Bruno
TI Correlations between objective and subjective sleep and circadian
markers in remitted patients with bipolar disorder
SO CHRONOBIOLOGY INTERNATIONAL
TC 7
Z9 7
SN EP 704
DI 10.3109/07420528.2014.895742
UT WOS:000336971900011
PM 24625313
ER

PT J Randler sample, explanation see methods
AU Di Milia, L
Randler, C
AF Di Milia, Lee
Randler, Christoph
TI The stability of the morning affect scale across age and gender
SO PERSONALITY AND INDIVIDUAL DIFFERENCES
TC 11
Z9 11
SN 0191-8869
PD JAN
PY 2013
VL 54
IS 2
BP 298
EP 301
DI 10.1016/j.paid.2012.08.031
UT WOS:000312926900029
ER

PT J Randler sample, explanation see methods

AU Randler, C
Ebenhoh, N
Fischer, A
Hochel, S
Schroff, C
Stoll, JC
Vollmer, C

AF Randler, Christoph
Ebenhoeh, Nadine
Fischer, Arian
Hoechel, Sandra
Schroff, Christina
Stoll, Julia Christin
Vollmer, Christian

TI Chronotype but not sleep length is related to salivary testosterone in young adult men

SO PSYCHONEUROENDOCRINOLOGY

Z9 24

SN 0306-4530

PD OCT

PY 2012

VL 37

IS 10

BP 1740

EP 1744

DI 10.1016/j.psyneuen.2012.02.008

UT WOS:000309097300015

PM 22425131

ER

PT J clinical
AU Suh, S
Nowakowski, S
Bernert, RA
Ong, JC
Siebern, AT
Dowdle, CL
Manber, R
AF Suh, Sooyeon
Nowakowski, Sara
Bernert, Rebecca A.
Ong, Jason C.
Siebern, Allison T.
Dowdle, Claire L.
Manber, Rachel
TI Clinical significance of night-to-night sleep variability in insomnia
SO SLEEP MEDICINE
TC 6
Z9 6
SN 1389-9457
PD MAY
PY 2012
VL 13
IS 5
BP 469

PT J adolescents
AU Vollmer, C
Randler, C
AF Vollmer, Christian
Randler, Christoph
TI Circadian preferences and personality values: Morning types prefer social values, evening types prefer individual values
SO PERSONALITY AND INDIVIDUAL DIFFERENCES
RI Vollmer, Christian/G-5084-2012; Vollmer, Christian/K-8253-2012
OI Vollmer, Christian/0000-0002-7763-7013
TC 12
Z9 12
SN 0191-8869
PD APR
PY 2012
VL 52
IS 6
BP 738
EP 743
DI 10.1016/j.paid.2012.01.001
UT WOS:000301623900016
ER

PT J --- meeting abstract
AU Chung, JK
Lee, KY
Jeong, SH
Kim, EJ
Ahn, YM
Joo, EJ
AF Chung, J. K.
Lee, K. Y.
Jeong, S. H.
Kim, E. J.
Ahn, Y. M.
Joo, E. J.
TI More eveningness in the patients with BP type II than BP type I disorder
SO BIPOLAR DISORDERS
CT 5th Biennial Conference of the
International-Society-for-Bipolar-Disorders
CY MAR 14-17, 2012
CL Istanbul, TURKEY
TC 0
Z9 0
SN 1398-5647
PD MAR
PY 2012
VL 14
SU 1
SI SI
BP 86
EP 86
UT WOS:000301531000197
ER

PT J adolescents
AU Vollmer, C
Randler, C
Di Milia, L
AF Vollmer, Christian
Randler, Christoph
Di Milia, Lee
TI Further Evidence for the Influence of Photoperiod at Birth on Chronotype
in a Sample of German Adolescents
SO CHRONOBIOLOGY INTERNATIONAL
RI Vollmer, Christian/K-8253-2012
OI Vollmer, Christian/0000-0002-7763-7013
TC 4
Z9 4
SN 0742-0528
PY 2012
VL 29
IS 10
BP 1345
EP 1351
DI 10.3109/07420528.2012.728656
UT WOS:000311837200006
PM 23130997
ER

PT J no CSM data
AU Ottoni, GL
Antoniolli, E
Lara, DR
AF Ottoni, Gustavo L.
Antoniolli, Eduardo
Lara, Diogo R.
TI Circadian Preference Is Associated With Emotional and Affective Temperaments
SO CHRONOBIOLOGY INTERNATIONAL
TC 10
Z9 11
SN 0742-0528
PY 2012
VL 29
IS 6
BP 786
EP 793
DI 10.3109/07420528.2012.679329
UT WOS:000305756700014
PM 22734579
ER

PT J Randler sample, explanation see methods

AU Randler, C
Stadler, L
Vollmer, C
Diaz-Morales, JF
AF Randler, Christoph
Stadler, Lisa
Vollmer, Christian
Francisco Diaz-Morales, Juan
TI Relationship Between Depressive Symptoms and Sleep Duration/Chronotype in Women
SO JOURNAL OF INDIVIDUAL DIFFERENCES F./0000-0002-3894-7908
TC 8
Z9 9
SN 1614-0001
PY 2012
VL 33
IS 3
BP 186
EP 191
DI 10.1027/1614-0001/a000089
UT WOS:000304974100007
ER

PT J --- binge drinkers, special group
AU Adan, A
AF Adan, Ana
TI Functional and dysfunctional impulsivity in young binge drinkers
SO ADICCIONES
RI Adan, Ana /B-3643-2011
OI Adan, Ana /0000-0002-3328-3452
TC 4
Z9 4
SN 0214-4840
PY 2012
VL 24
IS 1
BP 17
EP 22
UT WOS:000301024900003
PM 22508013
ER

PT J Randler sample, explanation see methods
AU Randler, C
Vollmer, C
AF Randler, Christoph
Vollmer, Christian
TI Epidemiological Evidence for the Bimodal Chronotype Using the Composite
Scale of Morningness
SO CHRONOBIOLOGY INTERNATIONAL
RI Vollmer, Christian/G-5084-2012; Vollmer, Christian/K-8253-2012
OI Vollmer, Christian/0000-0002-7763-7013
TC 4
Z9 4
SN 0742-0528
PY 2012
VL 29
IS 1
BP 1
EP 4
DI 10.3109/07420528.2011.635233
UT WOS:000298858800001
PM 22217095
ER

PT J --- adolescents
AU Vollmer, C
Schaal, S
Hummel, E
Randler, C
AF Vollmer, Christian
Schaal, Steffen
Hummel, Eberhard
Randler, Christoph
TI Association Among School-related, Parental and Self-related Problems and Morningness-Eveningness in Adolescents
SO STRESS AND HEALTH
RI Vollmer, Christian/G-5084-2012; Vollmer, Christian/K-8253-2012
OI Vollmer, Christian/0000-0002-7763-7013
TC 16
Z9 16
SN 1532-3005
PD DEC
PY 2011
VL 27
IS 5
BP 413
EP 419
DI 10.1002/smi.1393
UT WOS:000298298900008
ER

PT J
AU Lee, HJ --- overlap with other Korean studies
Kim, L
Kang, SG
Yoon, HK
Choi, JE
Park, YM
Kim, SJ
Kripke, DF
AF Lee, Heon-Jeong
Kim, Leen
Kang, Seung-Gul
Yoon, Ho-Kyoung
Choi, Jung-Eun
Park, Young-Min
Kim, Seog Ju
Kripke, Daniel F.
TI PER2 Variation is Associated with Diurnal Preference in a Korean Young
Population
SO BEHAVIOR GENETICS
TC 9
Z9 9
SN 0001-8244
PD MAR
PY 2011
VL 41
IS 2
BP 273
EP 277
DI 10.1007/s10519-010-9396-3
UT WOS:000287749700011
PM 20931356
ER

PT J adolescents
AU Lange, L
Randler, C
AF Lange, Luisa
Randler, Christoph
TI Morningness-eveningness and behavioural problems in adolescents
SO SLEEP AND BIOLOGICAL RHYTHMS
TC 20
Z9 20
SN 1446-9235
PD JAN
PY 2011
VL 9
IS 1
BP 12
EP 18
DI 10.1111/j.1479-8425.2010.00478.x
UT WOS:000286329900003
ER

PT J adolescents
AU Fromm, E
Horlebein, C
Meergans, A
Niesner, M
Randler, C
AF Fromm, Evantina
Horlebein, Christina
Meergans, Alexandra
Niesner, Marianne
Randler, Christoph
TI Evaluation of a dawn simulator in children and adolescents
SO BIOLOGICAL RHYTHM RESEARCH
TC 4
Z9 4
SN 0929-1016
PY 2011
VL 42
IS 5
BP 417
EP 425
DI 10.1080/09291016.2010.525851
UT WOS:000299786700005
ER

PT J no CSM data
AU Di Milia, L
Folkard, S
Hill, J
Walker, C
AF Di Milia, Lee
Folkard, Simon
Hill, Joanne
Walker, Curtis, Jr.
TI A Psychometric Assessment of the Circadian Amplitude and Phase Scale
SO CHRONOBIOLOGY INTERNATIONAL
TC 4
Z9 4
SN 0742-0528
PY 2011
VL 28
IS 1
BP 81
EP 87
DI 10.3109/07420528.2010.502603
UT WOS:000285582900010
PM 21182408
ER

PT J no CSM data
AU Besoluk, S
Onder, I
Deveci, I
AF Besoluk, Senol
Onder, Ismail
Deveci, Isa
TI Morningness-Eveningness Preferences and Academic Achievement of University Students
SO CHRONOBIOLOGY INTERNATIONAL
TC 40
Z9 40
SN 0742-0528
PY 2011
VL 28
IS 2
BP 118
EP 125
DI 10.3109/07420528.2010.540729
UT WOS:000286901900004
PM 21231873
ER

PT J --- sample overlap with Prat & Adan
AU Prat, G
   Adan, A
AF Prat, Gemma
   Adan, Ana
TI Influence of Circadian Typology on Drug Consumption, Hazardous Alcohol
   use, and Hangover Symptoms
SO CHRONOBIOLOGY INTERNATIONAL
RI Adan, Ana /B-3643-2011
OI Adan, Ana /0000-0002-3328-3452
TC 35
Z9 35
SN 0742-0528
PY 2011
VL 28
IS 3
BP 248
EP 257
DI 10.3109/07420528.2011.553018
UT WOS:000289000900007
PM 21452920
ER

PT J Randler sample, explanation see methods
AU Randler, C
   Kretz, S
AF Randler, Christoph
   Kretz, Stefanie
TI Assortative mating in morningness-eveningness
SO INTERNATIONAL JOURNAL OF PSYCHOLOGY
TC 10
Z9 10
SN 0020-7594
PY 2011
VL 46
IS 2
BP 91
EP 96
DI 10.1080/00207594.2010.518237
UT WOS:000288667900002
PM 22044180
ER

PT J
AU Randler, C adolescents
AF Randler, Christoph
TI Age and Gender Differences in Morningness-Eveningness During Adolescence
SO JOURNAL OF GENETIC PSYCHOLOGY
TC 22
Z9 22
SN 0022-1325
PY 2011
VL 172
IS 3
BP 302
EP 308
DI 10.1080/00221325.2010.535225
UT WOS:000300000500006
PM 21902008
ER

PT J
AU Randler, C **adolescents**
   Saliger, L
AF Randler, Christoph
   Saliger, Lena
TI Relationship between morningness-eveningness and temperament and
   character dimensions in adolescents
SO PERSONALITY AND INDIVIDUAL DIFFERENCES
TC 18
Z9 19
SN 0191-8869
PD JAN
PY 2011
VL 50
IS 2
BP 148
EP 152
DI 10.1016/j.paid.2010.09.016
UT WOS:000285233000004
ER

PT J
AU Randler, C **adolescents**
AF Randler, Christoph
TI Association between morningness-eveningness and mental and physical
health in adolescents
SO PSYCHOLOGY HEALTH & MEDICINE
TC 35
Z9 35
SN 1354-8506
EI 1465-3966
PY 2011
VL 16
IS 1
BP 29
EP 38
DI 10.1080/13548506.2010.521564
UT WOS:000286815400004
PM 21218362
ER

PT J – no CSM data reported
AU Lee, KY
Song, JY
Kim, SH
Kim, SC
Joo, EJ
Ahn, YM
Kim, YS
AF Lee, Kyu Young
Song, Joo Yun
Kim, Se Hyun
Kim, Seong Chan
Joo, Eun-Jeong
Ahn, Yong Min
Kim, Yong Sik
TI Association between CLOCK 3111T/C and preferred circadian phase in
Korean patients with bipolar disorder
SO PROGRESS IN NEURO-PSYCHOPHARMACOLOGY & BIOLOGICAL PSYCHIATRY
TC 23
Z9 24
SN 0278-5846
PD OCT 1
PY 2010
VL 34ER

PT J
AU Buschkens, J – **small sample**, special group
Graham, D
Cottrell, D
AF Buschkens, Jane
Graham, Deborah
Cottrell, David
TI Well-Being Under Chronic Stress: Is Morningness an Advantage?
SO STRESS AND HEALTH
RI Cottrell, David/I-6811-2013
OI Cottrell, David/0000-0002-2405-6362
TC 8
Z9 8
SN 1532-3005
PD OCT
PY 2010
VL 26
IS 4
BP 330
EP 340
DI 10.1002/smi.1300
UT WOS:000283274800007
ER

PT J
AU Randler, C **Randler sample, explanation see methods**
Schaal, S
AF Randler, Christoph
Schaal, Steffen
TI Morningness-eveningness, habitual sleep-wake variables and cortisol level
SO BIOLOGICAL PSYCHOLOGY
TC 45
Z9 45
SN 0301-0511
PD SEP
PY 2010
VL 85
IS 1
BP 14
EP 18
DI 10.1016/j.biopsycho.2010.04.006
UT WOS:000281182600002
PM 20450953
ER

PT J – **Randler sample, explanation see methods**
AU Randler, C
  Bausback, V
AF Randler, Christoph
  Bausback, Vanessa
TI Morningness-eveningness in women around the transition through menopause
  and its relationship with climacteric complaints
SO BIOLOGICAL RHYTHM RESEARCH
TC 10
Z9 10
SN 0929-1016
PY 2010
VL 41
IS 6
BP 415
EP 431
DI 10.1080/09291010903407631
UT WOS:000284408200001
ER

PT J – **no CSM data**
AU Clarisse, R
Le Floc'h, N
Kindelberger, C
Feunteun, P
AF Clarisse, Rene
Le Floc'h, Nadine
Kindelberger, Cecile
Feunteun, Patrick
TI DAILY RHYTHMICITY OF ATTENTION IN MORNING- VS. EVENING-TYPE ADOLESCENTS
AT BOARDING SCHOOL UNDER DIFFERENT PSYCHOSOCIOLOGICAL TESTING CONDITIONS
SO CHRONOBIOLOGY INTERNATIONAL
TC 14
Z9 14
SN 0742-0528
PY 2010
VL 27
IS 4
BP 826
EP 841
DI 10.3109/07420521003794051
UT WOS:000282027300008
PM 20560713
ER

PT J **adolescents**
AU Schmidt, S
Randler, C
AF Schmidt, Sarah
Randler, Christoph
TI Morningness-Eveningness and Eating Disorders in a Sample of Adolescent Girls
SO JOURNAL OF INDIVIDUAL DIFFERENCES
TC 19
Z9 20
SN 1614-0001
PY 2010
VL 31
IS 1
BP 38
EP 45
DI 10.1027/1614-0001/a000005
UT WOS:000274049300005
ER

PT J --- **adolescents**
AU Randler, C
Bilger, S
Diaz-Morales, JF
AF Randler, Christoph
Bilger, Sabrina
Francisco Diaz-Morales, Juan
TI Associations among Sleep, Chronotype, Parental Monitoring, and Pubertal Development among German Adolescents
SO JOURNAL OF PSYCHOLOGY
TC 39
Z9 40
SN 0022-3980
PD OCT
PY 2009
VL 143
IS 5
BP 509
EP 520
UT WOS:000271492000005
PM 19943401
ER

PT J --**adolescents**
AU Schneider, AM
Randler, C
AF Schneider, Anne-Marie
Randler, Christoph
TI Daytime sleepiness during transition into daylight saving time in adolescents: Are owls higher at risk?
SO SLEEP MEDICINE
TC 9
Z9 9
SN 1389-9457
PD OCT
PY 2009
VL 10
IS 9
BP 1047
EP 1050
DI 10.1016/j.sleep.2008.08.009
UT WOS:000271339000020
PM 19346161
ER

PT J
AU Caci, H – mean CSM not reported
Bouchez, J
Bayle, FJ
AF Caci, Herve
Bouchez, Jacques
Bayle, Franck J.
TI Inattentive Symptoms of ADHD Are Related to Evening Orientation
SO JOURNAL OF ATTENTION DISORDERS
TC 31
Z9 32
SN 1087-0547
PD JUL
PY 2009
VL 13
IS 1
BP 36
EP 41
DI 10.1177/1087054708320439
UT WOS:000276690500004
PM 19387003
ER

PT J – **Randler sample, explanation see methods**
AU Randler, C
AF Randler, Christoph
TI Validation of the full and reduced Composite Scale of Morningness
SO BIOLOGICAL RHYTHM RESEARCH
TC 39
Z9 39
SN 0929-1016
EI 1744-4179
PY 2009
VL 40
IS 5
BP 413
EP 423
DI 10.1080/09291010902731213
UT WOS:000269500500004
ER

PT J
AU Di Milia, L --- overlap with Natale & Di Milia (2011) and Bohle et al. (2001)
Bohle, P
AF Di Milia, Lee
Bohle, Philip
TI Morningness or Morning Affect? A Short Composite Scale of Morningness
SO CHRONOBIOLOGY INTERNATIONAL
RI Bohle, Philip/K-6397-2012
TC 20
Z9 20
SN 0742-0528
PY 2009
VL 26
IS 3
BP 494
EP 509
DI 10.1080/07420520902820954
UT WOS:000265291700008
PM 19360492
ER

PT J Randler sample, explanation see methods
AU Leonhard, C
   Randler, C
AF Leonhard, Catherine
   Randler, Christoph
TI In Sync with the Family: Children and Partners Influence the Sleep-Wake
   Circadian Rhythm and Social Habits of Women
SO CHRONOBIOLOGY INTERNATIONAL
TC 28
Z9 28
SN 0742-0528
PY 2009
VL 26
IS 3
BP 510
EP 525
DI 10.1080/07420520902821101
UT WOS:000265291700009
PM 19360493
ER

PT J --- adolescents
AU Randler, C
Frech, D
AF Randler, Christoph
Frech, Daniela
TI Young people's time-of-day preferences affect their school performance
SO JOURNAL OF YOUTH STUDIES
TC 23
Z9 23
SN 1367-6261
PY 2009
VL 12
IS 6
BP 653
EP 667
DI 10.1080/13676260902902697
UT WOS:000274745000004
ER

PT J Randler sample, explanation see methods
AU Schubert, E
Randler, C
AF Schubert, Eva
Randler, Christoph
TI Association between chronotype and the constructs of the
Three-Factor-Eating-Questionnaire
SO APPETITE
TC 25
Z9 26
SN 0195-6663
PD NOV
PY 2008
VL 51
IS 3
BP 501
EP 505
DI 10.1016/j.appet.2008.03.018
UT WOS:000259930900013
PM 18479778
ER

PT J Randler sample, explanation see methods
AU Randler, C
AF Randler, Christoph
TI EVENING TYPES AMONG GERMAN UNIVERSITY STUDENTS SCORE HIGHER ON SENSE OF HUMOR AFTER CONTROLLING FOR BIG FIVE PERSONALITY FACTORS
SO PSYCHOLOGICAL REPORTS
TC 4
Z9 4
SN 0033-2941
PD OCT
PY 2008
VL 103
IS 2
BP 361
EP 370
DI 10.2466/PR0.103.2.361-370
UT WOS:000261402800005
PM 19102459
ER

PT J Randler sample, explanation see methods
AU Randler, C
AF Randler, Christoph
TI Morningness-eveningness, sleep-wake variables and big five personality
   factors
SO PERSONALITY AND INDIVIDUAL DIFFERENCES
TC 45
Z9 45
SN 0191-8869
PD JUL
PY 2008
VL 45
IS 2
BP 191
EP 196
DI 10.1016/j.paid.2008.03.007
UT WOS:000257018200012
ER

PT J Randler sample, explanation see methods
AU Randler, C
AF Randler, Christoph
TI Psychometric properties of the German version of the Composite Scale of
Morningness
SO BIOLOGICAL RHYTHM RESEARCH
TC 43
Z9 43
SN 0929-1016
PD APR
PY 2008
VL 39
IS 2
BP 151
EP 161
DI 10.1080/09291010701424796
UT WOS:000253808000006
ER

PT J Randler sample, explanation see methods
AU Randler, C
AF Randler, Christoph
TI Morningness-eveningness and satisfaction with life
SO SOCIAL INDICATORS RESEARCH
TC 39
Z9 40
SN 0303-8300
PD APR
PY 2008
VL 86
IS 2
BP 297
EP 302
DI 10.1007/s11205-007-9139-x
UT WOS:000253529900007
ER

PT J special group, small sample
AU Monk, TH
Begley, AE
Billy, BD
Fletcher, ME
Germain, A
Mazumdar, S
Moul, DE
Shear, MK
Thompson, WK
Zarotney, JR

TI Sleep and circadian rhythms in spousally bereaved seniors
SO CHRONOBIOLOGY INTERNATIONAL
TC 10

PT J adolescents
AU Randler, C
AF Randler, Christoph
TI Differences in sleep and circadian preference between Eastern and Western German adolescents
SO CHRONOBIOLOGY INTERNATIONAL
TC 47
Z9 47
SN 0742-0528
PY 2008
VL 25
IS 4
BP 565
EP 575
DI 10.1080/07420520802257794
UT WOS:000257544100006
PM 18622816
ER

PT J no CSM data
AU Tonetti, L
   Fabbri, M
   Natale, V
AF Tonetti, Lorenzo
   Fabbri, Marco
   Natale, Vincenzo
TI Sex difference in sleep-time preference and sleep need: A
   cross-sectional survey among Italian pre-adolescents, adolescents, and
   adults
SO CHRONOBIOLOGY INTERNATIONAL
TC 92
Z9 92
SN 0742-0528
EI 1525-6073
PY 2008
VL 25
IS 5
BP 745
EP 759
DI 10.1080/07420520802394191
UT WOS:000259039700006
PM 18780201
ER

PT J no CSM data
AU Di Milia, L
Wikman, R
Smith, P
AF Di Milia, Lee
Wikman, Renee
Smith, Peter
TI Additional psychometric evidence and construct validity for a revised preferences scale of morningness
SO CHRONOBIOLOGY INTERNATIONAL
TC 12
Z9 12
SN 0742-0528
EI 1525-6073
PY 2008
VL 25
IS 5
BP 776
EP 787
DI 10.1080/07420520802391098
UT WOS:000259039700008
PM 18780203
ER

PT J adolescents
AU Randler, C
AF Randler, Christoph
TI Morningness-Eveningness Comparison in Adolescents from Different
Countries around the World
SO CHRONOBIOLOGY INTERNATIONAL
TC 64
Z9 64
SN 0742-0528
PY 2008
VL 25
IS 6
BP 1017
EP 1028
DI 10.1080/07420520802551519
UT WOS:000260850600012
PM 19005902
ER

PT J adolescents
AU Diaz-Morales, JF
Randler, C
AF Diaz-Morales, Juan Francisco
Randler, Christoph
TI Morningness-Eveningness Among German and Spanish Adolescents 12-18 Years
SO EUROPEAN PSYCHOLOGIST
RI Diaz-Morales, Juan F./A-7797-2010
OI Diaz-Morales, Juan F./0000-0002-3894-7908
TC 19
Z9 19
SN 1016-9040
PY 2008
VL 13
IS 3
BP 214
EP 221
DI 10.1027/1016-9040.13.3.214
UT WOS:000260571200007
ER

PT J Review
AU Cavallera, GM
Giudici, S
AF Cavallera, G. M.
Giudici, S.
TI Morningness and eveningness personality: A survey in literature from
1995 up till 2006
SO PERSONALITY AND INDIVIDUAL DIFFERENCES
TC 60
Z9 60
SN 0191-8869
PD JAN
PY 2008
VL 44
IS 1
BP 3
EP 21
DI 10.1016/j.paid.2007.07.009
UT WOS:000251045000001
ER

PT J Randler sample, explanation see methods
AU Randler, C
AF Randler, Christoph
TI Differences between smokers and nonsmokers in morningness-eveningness
SO SOCIAL BEHAVIOR AND PERSONALITY
TC 19
Z9 20
SN 0301-2212
PY 2008
VL 36
IS 5
BP 673
EP 679
DI 10.2224/sbp.2008.36.5.673
UT WOS:000259459000010
ER

PT J Review
AU Randler, C
AF Randler, Christoph
TI Gender differences in morningness-eveningness assessed by self-report
   questionnaires: A meta-analysis
SO PERSONALITY AND INDIVIDUAL DIFFERENCES
TC 80
Z9 80
SN 0191-8869
PD NOV
PY 2007
VL 43
IS 7
BP 1667
EP 1675
DI 10.1016/j.paid.2007.05.004
UT WOS:000250979500003
ER

PT J Randler sample, explanation see methods
AU Randler, C
Diaz-Morales, JF
AF Randler, Christoph
Diaz-Morales, Juan Francisco
TI Morningness in German and Spanish students: A comparative study
SO EUROPEAN JOURNAL OF PERSONALITY
TC 40
Z9 40
SN 0890-2070
PD JUN
PY 2007
VL 21
IS 4
BP 419
EP 427
DI 10.1002/per.632
UT WOS:000247380700003
ER

PT J no CSM data
AU Hur, YM
AF Hur, Yoon-Mi
TI Stability of genetic influence on morningness-eveningness: a
cross-sectional examination of South Korean twins from preadolescence to
young adulthood
SO JOURNAL OF SLEEP RESEARCH
TC 67
Z9 67
SN 0962-1105
PD MAR
PY 2007
VL 16
IS 1
BP 17
EP 23
DI 10.1111/j.1365-2869.2007.00562.x
UT WOS:000244277500003
PM 17309759
ER

PT J children
AU Diaz-Morales, JF
de Leon, CD
Sorroche, MG
AF Diaz-Morales, Juan Francisco
de Leon, Celeste Davila
Gutierrez Sorroche, Maria
TI Validity of the morningness-eveningness scale for children among Spanish
adolescents
SO CHRONOBIOLOGY INTERNATIONAL
TC 35
Z9 35
SN 0742-0528
PY 2007
VL 24
IS 3
BP 435
EP 447
DI 10.1080/07420520701420659
UT WOS:000247726400005
PM 17612943
ER

PT J --- overlap with other studies
AU Lee, HJ
Paik, JW
Kang, SG
Lim, SW
Kim, L
AF Lee, Heon-Jeong
Paik, Jong-Woo
Kang, Seung-Gul
Lim, Se-Won
Kim, Leen
TI Allelic variants interaction of clock gene and g-protein beta 3 subunit gene with diurnal preference
SO CHRONOBIOLOGY INTERNATIONAL
TC 17
Z9 17
SN 0742-0528
PY 2007
VL 24
IS 4
BP 589
EP 597
DI 10.1080/07420520701534632
UT WOS:000249247300002
PM 17701674
ER

PT J --- duplicate sample with Caci et al (2005)
AU Adan, A
Caci, H
Prat, G
AF Adan, A
Caci, H
Prat, G
TI Reliability of the Spanish version of the Composite Scale of Morningness
SO EUROPEAN PSYCHIATRY
TC 24
Z9 25
SN 0924-9338
PD NOV
PY 2005
VL 20
IS 7
BP 503
EP 509
DI 10.1016/j.eurpsy.2005.01.003
UT WOS:000233712600007
PM 16310682
ER

PT J no CSM data
AU Di Milia, L
Smith, PA
Folkard, S
AF Di Milia, L
Smith, PA
Folkard, S
TI A validation of the revised circadian type inventory in a working sample
SO PERSONALITY AND INDIVIDUAL DIFFERENCES
Z9 26
SN 0191-8869
PD NOV
PY 2005
VL 39
IS 7
BP 1293
EP 1305
DI 10.1016/j.paid.2005.04.012
UT WOS:000233122200012
ER

PT J adolescence

AU Natale, V
Sansavini, A
Trombini, E
Esposito, MJ
Alessandroni, R
Faldella, G
AF Natale, V
Sansavini, A
Trombini, E
Esposito, MJ
Alessandroni, R
Faldella, G
TI Relationship between preterm birth and circadian typology in adolescence
SO NEUROSCIENCE LETTERS
TC 7
Z9 8
SN 0304-3940
PD JUL 1
PY 2005
VL 382
IS 1-2
BP 139
EP 142
DI 10.1016/j.neulet.2005.03.004
UT WOS:000229661900027
PM 15911137
ER

PT J special group selected
AU Willis, TA
O'Connor, DB
Smith, L
AF Willis, TA
O'Connor, DB
Smith, L
TI The influence of morningness-eveningness on anxiety an cardiovascular responses to stress
SO PHYSIOLOGY & BEHAVIOR
TC 26
Z9 26
SN 0031-9384
PD JUN 2
PY 2005
VL 85
IS 2
BP 125
EP 133
DI 10.1016/j.physbeh.2005.03.013
UT WOS:000229805900006
PM 15924909
ER

PT J: duplicate sample to Caci et al. (2005)
AU Caci, H
Mattei, V
Bayle, FJ
Nadalet, L
Dossios, C
Robert, P
Boyer, P
AF Caci, H
Mattei, V
Bayle, FJ
Nadalet, L
Dossios, C
Robert, P
Boyer, P
TI Impulsivity but not venturesomeness is related to morningness
SO PSYCHIATRY RESEARCH
TC 50
Z9 50
SN 0165-1781
PD APR 30
PY 2005
VL 134
IS 3
BP 259
EP 265
DI 10.1016/j.psychres.2004.02.019
UT WOS:000229867000006
PM 15892985
ER

PT J no CSM data
AU Di Milia, L
AF Di Milia, L
TI A psychometric evaluation and validation of the preferences scale
SO CHRONOBIOLOGY INTERNATIONAL
TC 9
Z9 9
SN 0742-0528
PY 2005
VL 22
IS 4
BP 679
EP 693
DI 10.1080/07420520500180454
UT WOS:000232137700006
PM 16147899
ER

PT J children
AU Caci, H
Robert, P
Dossios, C
Boyer, P
AF Caci, H
Robert, P
Dossios, C
Boyer, P
TI Morningness-Eveningness for Children Scale: psychometric properties and
month of birth effect
SO ENCEPHALE-REVUE DE PSYCHIATRIE CLINIQUE BIOLOGIQUE ET THERAPEUTIQUE
TC 32
Z9 VL 31:000228261500007
PM

PT J no CSM means
AU Caci, H
Robert, P
Boyer, P
AF Caci, H
Robert, P
Boyer, P
TI Novelty seekers and impulsive subjects are low in morningness
SO EUROPEAN PSYCHIATRY
SN 0924-9338
PD APR
PY 2004
VL 19
IS 2
BP 79
EP 84
DI 10.1016/j.eurpsy.2003.09.007
UT WOS:000220858300003
PM 15051106
ER

PT J
AU Monk, TH --- excluded no data
Buysse, DJ
Potts, JM
DeGrazia, JM
Kupfer, DJ
AF Monk, TH
Buysse, DJ
Potts, JM
DeGrazia, JM
Kupfer, DJ
TI Morningness-eveningness and lifestyle regularity
SO CHRONOBIOLOGY INTERNATIONAL
TC 61
Z9 62
SN 0742-0528
PY 2004
VL 21
IS 3
BP 435
EP 443
DI 10.1081/CBI-120038614
UT WOS:000223157100009
PM 15332448
ER

PT J small sample special group
AU Natale, V
Martoni, M
Cicogna, P
AF Natale, V
Martoni, M
Cicogna, P
TI Effects of circadian typology on sleep-wake behavior of air traffic controllers
SO PSYCHIATRY AND CLINICAL NEUROSCIENCES
TC 9
Z9 10
SN 1323-1316
PD OCT
PY 2003
VL 57
IS 5
BP 539
EP 541
DI 10.1046/j.1440-1819.2003.01160.x
UT WOS:000185084400013
PM 12950710
ER

PT J no CSM data
AU Natale, V
Cicogna, P
AF Natale, V
Cicogna, P
TI Morningness-eveningness dimension: is it really a continuum?
SO PERSONALITY AND INDIVIDUAL DIFFERENCES
TC 112
Z9 113
SN 0191-8869
PD APR 5
PY 2002
VL 32
IS 5
BP 809
EP 816
DI 10.1016/S0191-8869(01)00085-X
UT WOS:000174990800004
ER

PT J no CSM data
AU Koscec, A
Radosevic-Vidacek, B
Kostovic, M
AF Koscec, A
Radosevic-Vidacek, B
Kostovic, M
TI Morningness-eveningness across two student generations: would two decades make a difference?
SO PERSONALITY AND INDIVIDUAL DIFFERENCES
CT Psychological Conference on XIV Ramiro Bujas Days
CY DEC, 1999
CL ZAGREB, CROATIA
TC 7
Z9 7
SN 0191-8869
PD SEP
PY 2001
VL 31
IS 4
BP 627
EP 638
UT WOS:000170080800015
ER

PT J CSM mean and age not reported
AU Natale, V
   Alzani, A
AF Natale, V
   Alzani, A
TI Additional validity evidence for the composite scale of morningness
SO PERSONALITY AND INDIVIDUAL DIFFERENCES
TC 35
Z9 37
SN 0191-8869
PD JAN 19
PY 2001
VL 30
IS 2
BP 293
EP 301
DI 10.1016/S0191-8869(00)00046-5
UT WOS:000165679500010
ER

PT J no CSM data
AU Guardia, J
Adan, A
AF Guardia, J
Adan, A
TI Confirmatory factor analysis applied to Matthews adjective checklist of self-reported activation: Effect of time of day
SO QUALITY & QUANTITY
TC 3
Z9 3
SN 0033-5177
PD FEB
PY 1997
VL 31
IS 1
BP 95
EP 106
DI 10.1023/A:1004242119822
UT WOS:A1997WN39300006
ER

*Baek J.H., Kim J.S., Kim M.J., Ryu S., Lee K., Ha K., Hong K.S.,"Lifetime Characteristics of Evening-Preference and Irregular Bed-Rise Time Are Associated With Lifetime Seasonal Variation of Mood and Behavior: Comparison Between Individuals With Bipolar Disorder and Healthy Controls",2014,"Behavioral Sleep Medicine",,,,"","",14,,10.1080/15402002.2014.974179,"http://www.scopus.com/inward/record.url?eid=2-s2.0-84909942525&partnerID=40&md5=fd21fae9f16eb08434fb859cf1f8982d","Department of Psychiatry, Sungkyunkwan University School of Medicine Samsung Medical Center --- **duplicate sample to Bae et al.***

Smith C.S., Reilly C., Midkiff K.,"Evaluation of Three Circadian Rhythm Questionnaires With Suggestions for an Improved Measure of Morningness",1989,"Journal of Applied Psychology","74","5",,"728","738",,376,,"http://www.scopus.com/inward/record.url?eid=2-s2.0-0024747515&partnerID=40&md5=b7365e9fe0192dcadc99b1d4e1463a87","Bowling Green State University, United States","Smith, C.S., Bowling Green State University, United States ***--- no age given***

*Morales J.F.D., Sanchez-Lopez M.P., Thorne C.,"The composite and the preferences scales of morningness in peruvian university students [La escala de preferencias y la escala compuesta de matutinidad en universitarios peruanos]",2005,"Interamerican Journal of Psychology","39","2",,"203","210",,,,"http://www.scopus.com/inward/record.url?eid=2-s2.0-77954972993&partnerID=40&md5=14e2ae4c83c029ee35952adb94cef873","Departamento de Psicología Diferencial y Psicología del Trabajo, Facultad de Psicología, Campus de Somosaguas, s/n, 28223, Madrid, Spain --- **duplicate sample***

Ong J.C., Huang J.S., Kuo T.F., Manber R.,"Characteristics of insomniacs with self-reported morning and evening chronotypes",2007,"Journal of Clinical Sleep Medicine","3","3",,"289","294",,41,,"http://www.scopus.com/inward/record.url?eid=2-s2.0-34248596197&partnerID=40&md5=7a254f3b4ead8d8757a71b29593dbe32","Department of Psychiatry and Behavioral Sciences, Stanford University, Stanford, CA, United States ***clinical***

Horzum MB, Randler C, Masal E, Beşoluk Ş, Önder İ, Vollmer C. Morningness-eveningness and the environment hypothesis - A cross-cultural comparison of Turkish and German adolescents. Chronobiol Int. 2015 Jul;32(6):814-821. Epub 2015 Jun 10. PubMed PMID: 26061589 **adolescents**

Bei B, Ong JC, Rajaratnam SM, Manber R. Chronotype and Improved Sleep Efficiency Independently Predict Depressive Symptom Reduction after Group Cognitive Behavioral Therapy for Insomnia. J Clin Sleep Med. 2015 Mar 16. pii: jc-00065-15. [Epub ahead of print] PubMed PMID: 25845891. **clinical**

Schaal S., Peter M., Randler C.,"Morningness-eveningness and physical activity in adolescents",2010,"International Journal of Sport and Exercise Psychology","8","2",,"147","159",,9,,"http://www.scopus.com/inward/record.url?eid=2-s2.0-77957722328&partnerID=40&md5=5ef279c4afbd909bc0760b85c3388320","University of Bamberg, Germany --- ***adolescents***

*Mansour H., Tobar S., Fathi W., Ibrahim I., Wood J., Elassy M., Elsayed H., Yassin A., Salah H., Eissa A., El-Boraie H., El-Boraie O., Dobea A., Osama H., Gomaa Z., El-Bahaei W., Monk T.H., Nimgaonkar V.L.,"Arabic versions of the sleep timing questionnaire and the composite scale of morningness",2015,"Asian Journal of Psychiatry","13",,,"48","51",,,10.1016/j.ajp.2014.10.004,"http://www.scopus.com/inward/record.url?eid=2-s2.0-84926316754&partnerID=40&md5=928f35e6fdab6b7a288e5e1c044d90b8","Department of Psychiatry, University of Pittsburgh School of Medicine, Western Psychiatric Institute and Clinic, Pittsburgh, PA, United States --**- no data available on CSM on submission date***

Chung J.K., Lee K.Y., Kim S.H., Kim E.-J., Jeong S.H., Jung H.Y., Choi J.-E., Ahn Y.M., Kim Y.S., Joo E.-J.,"Circadian rhythm characteristics in mood disorders: Comparison among bipolar I disorder, bipolar II disorder and recurrent major depressive disorder",2012,"Clinical Psychopharmacology and Neuroscience","10","2",,"110","116",,9,,"http://www.scopus.com/inward/record.url?eid=2-s2.0-84865806331&partnerID=40&md5=08d070cbc4560d75fc757e0d2b5792bd","Department of Neuropsychiatry, Eulji General Hospital, Eulji University School of Medicine, 14, Hangeulbiseong-ro, Nowon-gu, Seoul 139-711, South Korea-- ***clinical***

*Levandovski R., Sasso E., Hidalgo M.P.,"Chronotype: A review of the advances, limits and applicability of the main instruments used in the literature to assess human phenotype [Cronotipo: Uma revisão dos avanços, limites e aplicabilidade dos principais instrumentos utilizados na literatura para avaliar o fenótipo humano]",2013,"Trends in Psychiatry and Psychotherapy","35","1",,"3","11",,3,10.1590/S2237-60892013000100002,"http://www.scopus.com/inward/record.url?eid=2-s2.0-84878447122&partnerID=40&md5=53356e0c0cca8380ee7652a6bee69499","Laboratório de Cronobiologia, Hospital de Clínicas de Porto Alegre HCPA), Universidade Federal do Rio Grande do Sul (UFRGS), Porto Alegre, RS, Brazil --- **review***

*Perea C.S., Nino C.L., Lopez-Leon S., Gutierrez R., Ojeda D., Arboleda H., Camargo A., Adan A., Forero D.A.,"Study of a functional polymorphism in the PER3 gene and diurnal preference in a Colombian sample",2014,"Open Neurology Journal","8","1",,"7","10",,2,10.2174/1874205X01408010007,"http://www.scopus.com/inward/record.url?eid=2-s2.0-84900868168&partnerID=40&md5=a1da0f114320ae424fdb1eb8a0658cbe","Laboratory of NeuroPsychiatric Genetics, Biomedical Sciences Research Group, School of Medicine, Universidad Antonio Nariño. Bogotá, Colombia --- **overlap with Ojeda et al.***

Duarte J., Nelas P., Chaves C., Ferreira M., Coutinho E., Cunha M.,"Sleep-wake patterns and their influence on school performance in Portuguese adolescents",2014,"Atencion Primaria","46","S5",,"160","164",,,10.1016/S0212-6567(14)70085-X,"http://www.scopus.com/inward/record.url?eid=2-s2.0-84919658342&partnerID=40&md5=190ac2568f16b21e9ecce879a80e3a72","Escola Superior de Saúde de Viseu, Instituto Politécnico de Viseu, Viseu, Portugal","Duarte, J., Escola Superior de Saúde de Viseu, Instituto Politécnico de Viseu, Viseu, Portugal --- ***adolescents***
